# Therapeutic potential of combined BRAF/MEK blockade in *BRAF*-wild type preclinical tumor models

**DOI:** 10.1186/s13046-018-0820-5

**Published:** 2018-07-09

**Authors:** Anais Del Curatolo, Fabiana Conciatori, Ursula Cesta Incani, Chiara Bazzichetto, Italia Falcone, Vincenzo Corbo, Sabrina D’Agosto, Adriana Eramo, Giovanni Sette, Isabella Sperduti, Teresa De Luca, Mirko Marabese, Senji Shirasawa, Ruggero De Maria, Aldo Scarpa, Massimo Broggini, Donatella Del Bufalo, Francesco Cognetti, Michele Milella, Ludovica Ciuffreda

**Affiliations:** 1 Medical Oncology 1, IRCCS Regina Elena National Cancer Institute, Rome, Italy; 2 ARC-Net Research Centre and Department of Pathology, University of Verona, Verona, Italy; 3 University of Rome “La Sapienza”, Rome, Italy; 4 Department of Hematology, Oncology and Molecular Medicine, Istituto Superiore di Sanità, Rome, Italy; 5 Biostatistics, IRCCS Regina Elena National Cancer Institute, Rome, Italy; 6 Preclinical Models and New Therapeutic Agents Unit, IRCCS Regina Elena National Cancer Institute, Rome, Italy; 7 Laboratory of Molecular Pharmacology, Department of Oncology, IRCCS - Istituto di Ricerche Farmacologiche “Mario Negri”, Milan, Italy; 8 Central Research Institute for Advanced Molecular Medicine, Fukuoka University, Fukuoka, Japan; 9 Institute of General Pathology, Catholic University of the Sacred Heart, Rome, Italy

**Keywords:** MAPK, BRAF, MEK, Combination therapy, Paradoxical effect

## Abstract

**Background:**

Mounting evidence suggests that RAF-mediated MEK activation plays a crucial role in paradox MAPK (re)activation, leading to resistance and therapeutic failure with agents hitting a single step along the MAPK cascade.

**Methods:**

We examined the molecular and functional effects of single and combined BRAF (dabrafenib), pan-RAF (RAF265), MEK (trametinib) and EGFR/HER2 (lapatinib) inhibition, using Western Blot and conservative isobologram analysis to assess functional synergism, and explored genetic determinants of synergistic interactions. Immunoprecipitation based assays were used to detect the interaction between BRAF and CRAF. The Mann-Whitney U test was used for comparing quantitative variables.

**Results:**

Here we demonstrated that a combination of MEK and BRAF inhibitors overcomes paradoxical MAPK activation (induced by BRAF inhibitors) in *BRAF*-wt/*RAS*-mut NSCLC and PDAC in vitro. This results in growth inhibitory synergism, both in vitro and in vivo, in the majority (65%) of the cellular models analyzed, encompassing cell lines and patient-derived cancer stem cells and organoids. However, RAS mutational status is not the sole determinant of functional synergism between RAF and MEK inhibitors, as demonstrated in KRAS isogenic tumor cell line models. Moreover, in EGFR-driven contexts, paradoxical MAPK (re)activation in response to selective BRAF inhibition was dependent on EGFR family signaling and could be offset by simultaneous EGFR/HER-2 blockade.

**Conclusions:**

Overall, our data indicate that RAF inhibition-induced paradoxical MAPK activation could be exploited for therapeutic purposes by simultaneously targeting both RAF and MEK (and potentially EGFR family members) in appropriate molecular contexts. *KRAS* mutation per se does not effectively predict therapeutic synergism and other biomarkers need to be developed to identify patients potentially deriving benefit from combined BRAF/MEK targeting.

**Electronic supplementary material:**

The online version of this article (10.1186/s13046-018-0820-5) contains supplementary material, which is available to authorized users.

## Background

The Mitogen-Activated Protein Kinase (MAPK) pathway is a key signaling pathway involved in the physiologic regulation of cell growth, survival, differentiation, apoptosis and migration [1]. Aberrant activation of the MAPK pathway has been implicated in the pathogenesis of many human diseases, including cancer, in which MAPK activation may stem from either genetic aberrations targeting *RAS*, B Rapidly Accelerated Fibrosarcoma (*BRAF)*, or Mitogen-activated protein kinase kinase (*MEK)* directly or dysregulation of upstream acting Receptor Tyrosine Kinases (RTK) [2, 3]. MAPK activation is finely regulated at different levels; moreover, in addition to “inter-pathway” crosstalks operating at multiple levels between MAPK and other signaling cascades (e.g. PhosphoInositide3-Kinase (PI3K)/ Protein kinase B (AKT)/ mammalian Target of Rapamycin (mTOR) [4, 5]) “intra-pathway” feedback loops regulate Extracellular-signal-Regulated Kinase (ERK) activity through phosphorylation, intracellular localization, and complex formation [6–8]. As a result, inhibition of a single step (either BRAF or MEK) of the cascade has met with limited clinical success [9], presumably because of the interruption of negative feedback loops leading to downstream pathway (re)activation. Indeed, in some genetic contexts, selective BRAF inhibition has been linked to paradoxical MAPK activation, a phenomenon attributed to the ability of BRAF inhibitors to activate RAF signaling by promoting CRAF-BRAF dimerization, in *BRAF* wild-type cells [10–12].

Over the past few years, a vertical combination of BRAF and MEK inhibitors (dabrafenib/trametinib or vemurafenib/cobimetinib) has demonstrated striking clinical efficacy and has become a standard of care in patients with *BRAF*-mut melanoma and lung cancer [13–16], although the molecular mechanisms by which combined BRAF/MEK inhibition results in synergistic anti-tumor activity are still not completely clear and the same combinations have not met with the same therapeutic success in other *BRAF*-mut cancer type of different histological origin [17]. On the other hand, RAF activation has been shown to be crucial to RAS-mediated transformation and its inhibition, by either isoform-selective or non-selective inhibitors, appears to be essential for effective downstream MEK/ERK inhibition [18] and strikingly synergizes with MEK inhibition in controlling tumor growth and overcoming resistance, particularly in KRAS mutation-driven contexts [19, 20].

Here we analyzed the molecular and functional effects of combined BRAF/MEK inhibition in cell line and patient-derived preclinical models of Non-Small Cell Lung Cancer (NSCLC) and Pancreatic Ductal AdenoCarcinoma (PDAC) and found that selective BRAF inhibition causes paradoxical MAPK activation that could be reversed by simultaneous MEK blockade; functionally, a combination of dabrafenib (selective BRAF inhibitor) and trametinib (selective MEK inhibitor) achieved strikingly synergistic in vitro tumor growth inhibition in a proportion of the models assessed; however, RAS mutational status appears not to be sole determinant of functional synergism between RAF and MEK inhibitors. Indeed, in Epidermal Growth Factor Receptor (EGFR)-family driven models, paradoxical MAPK reactivation appears to be sustained by RTK signaling and may be reversed by EGFR/Human Epidermal Growth Factor Receptor 2 (HER2)- blockade.

## Methods

### Cell lines

HCC827 cell lines were obtained from the American Type Culture Collection (ATCC). Isogenic cells lines HCT116 (HK2–6 and HKE-3) were kindly provided by Dr. Shirasawa and performed by gene targeting technique [21]; NCI-H1299 cell lines and relative clones (H1299 K4, D2, C2, V9) were kindly donated by Dr. Broggini [22]. Lung Cancer Stem Cells (LCSC) were generated in vitro as previously reported [23, 24].

Cell lines were routinely maintained in RPMI 1640 or DMEM medium supplemented with 10% fetal bovine serum (FBS), 2 mM L-glutamine, and antibiotics in a humidified atmosphere with 5% CO_2_ at 37 °C.

### Drugs treatment and cell proliferation assay

Trametinib (GSK1120212), dabrafenib (GSK2118436) and lapatinib (Tyverb) were kindly provided by GlaxoSmithKline (Brentford, Middlesex, UK). Trametinib and lapatinib were dissolved in DMSO as a 1 mM stock solution and stored at − 20 °C, dabrafenib was dissolved in DMSO as a 10 mM stock solution and stored at − 20 °C. RAF265 (CHIR-265, R265) was obtained from Novartis Pharma (Basel, Switzerland) and dissolved in DMSO as a 10 mM stock solution and stored at − 20 °C. The final concentration of both drugs was obtained by dilution with culture medium. Effects on cell growth in response to different treatments were monitored by Crystal Violet assay [24]. For Crystal Violet assay, a fixed number of tumor cells were dispensed into 24-wells (NEST Biotechnology), and the following day cells were treated at indicated concentrations of drugs [24].

### Xenograft experiments

Procedures relative to animal use and care were authorized and certified by Italian Minister of Health (decree n. 67/97A, protocol 2560/97, Rome Health Service Unit). Regina Elena National Cancer Institute and animal care Unit approved the procedures involving animals (species, quality and number of animals, discomfort/distress/pain of animals, anaesthesia and euthanasia).

5 × 10^6^ MiaPaCa2 cells were intramuscularly injected into immunodeficient athymic mice (6–8 week-old female). Four groups of animals with similar tumor volume were created when the tumors reached palpability. The following treatments were administered for two weeks: 1) daily vehicle administration; 2) daily oral administration with 0.2 mg/Kg trametinib; 3) daily oral administration with 30 mg/Kg dabrafenib; 4) combination treatment (0.2 mg/Kg trametinib + 30 mg/Kg dabrafenib). 6 animals composed each group. Tumor weight (in mg) was estimated daily based on the measurement of the longest perpendicular diameters, as previously described [24]. Animals were also daily monitored for food consumption, body-weight, and behavior. Animals were sacrificed 27 days after tumor cell injection. Differences between treatment groups were analyzed by 2-tailed Student’s t test for unpaired samples; statistical significance was set at *p* < 0.05.

### Human pancreatic tumor and normal organoid culture: Isolation, culture and proliferation assay

Human neoplastic organoid cultures were established from resected PDAC specimens. Specimens from pancreatic resections were digested enzymatically with collagenase/dispase dissociation and then plated in Matrigel to generate pancreas organoid cultures, in human complete medium [25]. PDAC organoids were then maintained in human complete medium. For the 50% of cell growth inhibition (IC_50_) analysis, organoids were dissociated into single cells by first triturating them in media through a fire-polished glass pipette, and then by enzymatic dissociation with 2 mg/ml dispase dissolved in TrypLE (Life Technologies). Cells were counted and diluted to 10 cells/μl in a mixture of complete media, Rho Kinase inhibitor Y-27632 (10.5 μM final concentration, Sigma) and Growth factor-reduced Matrigel (GFR-Matrigel, 10% final concentration). 100 μl of this mixture (1000 cells/well) was plated in 96-well plates (Nunc), whose wells had been previously coated with a bed of GFR-Matrigel to prevent attachment of the cells to the bottom of the plate. Cell viability in response to the different drugs was measured using the CellTiter-Glo assay (Promega Corporation) and Synergy 4 reader (Biotek).

### Sanger sequencing for cell lines and organoids

DNA was extracted from cell lines using the QIAamp DNA mini kit (Qiagen) and quantified with the Nanodrop Spectrophotometer (Thermo). Primers for amplification and sequencing of exon 2 of the KRAS gene (Fw: aggcctgctgaaaatgactgaata and Rw: ctgtatcaaagaatggtcctgcac) and of exon 15 of the BRAF gene (Fw: tcataatgcttgctctgatagga and Rv: ggccaaaaatttaatcagtgga), PCR products were purified using the Nucleospin kit and sequenced by capillary electrophoresis using the BigDye Terminator v3.1 cycle sequencing kit on 3130 Genetic Analyzer (Applied Biosystem). Sequence traces were analyzed using the Clustal W program (https://embnet.vital-it.ch/software/ClustalW.html). For organoids Sanger Sequencing see https://www.ncbi.nlm.nih.gov/pmc/articles/PMC4334572/#SD15 [25].

### Western blot (WB) analysis

**Fig. 1:**
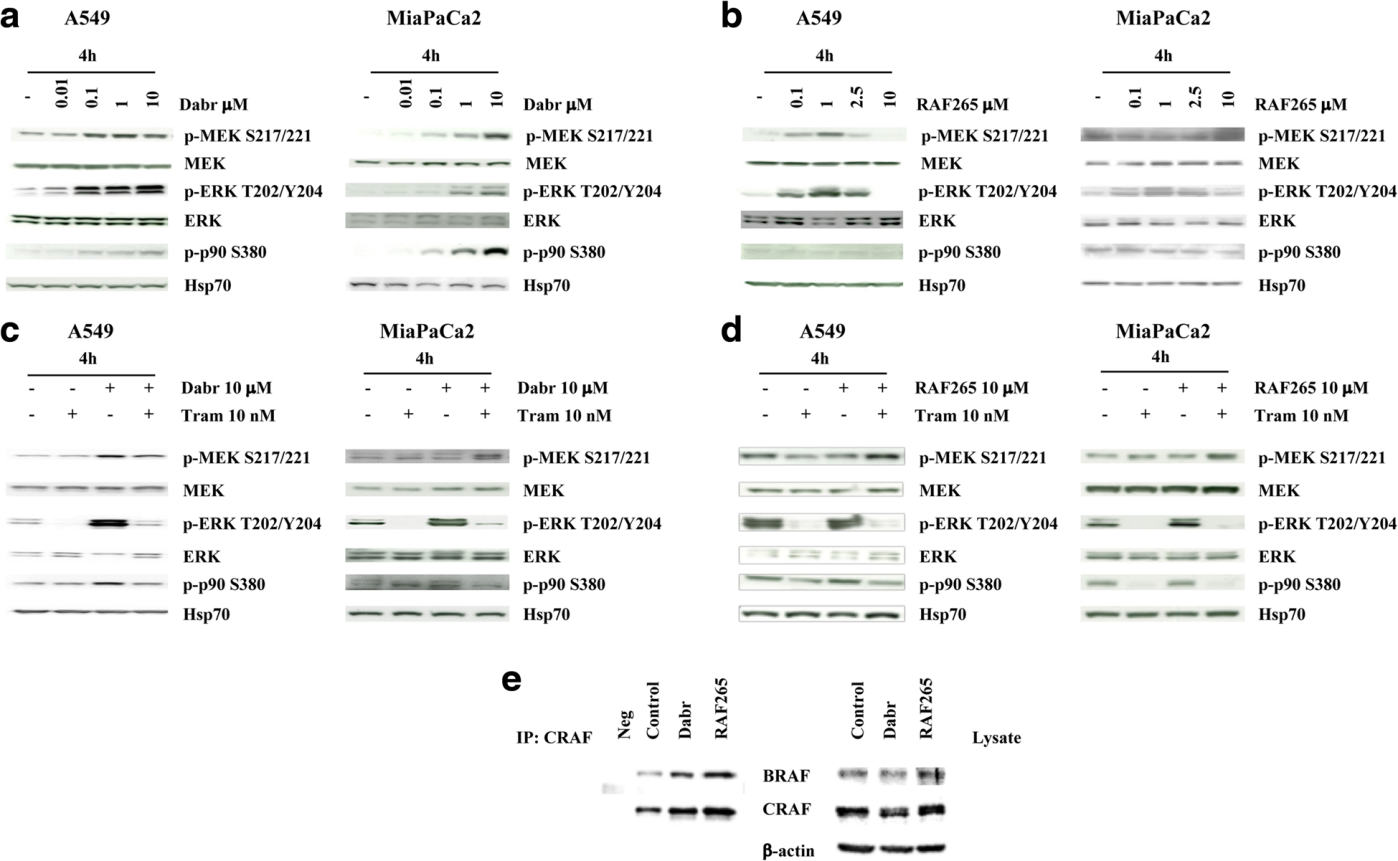
Molecular analysis in *KRAS*-mut lung and pancreatic cell lines. **a**-**d**. Lung cancer cell line A549 and pancreatic cancer cell line MiaPaCa2 were treated with dabrafenib (**a**) and RAF265 (**b**) alone or in combination with trametinib (**c**-**d**) for 4 h at the indicated doses. The cells were lysed and analyzed by Western Blotting using antibodies specific for the indicated proteins. Western blot with Hsp-70 specific antibody is shown as protein loading and blotting control. **e** A549 cells were treated with dabrafenib (10 μM) or RAF265 (10 μM) for 4 h. Endogenous CRAF was immunoprecipitated and the immunocomplexes were blotted for BRAF or CRAF. BRAF and CRAF levels in total cell lysates are also shown

For western blotting, total cell lysates were prepared as described previously [26]. The proteins were fractionated by SDS-polyacrylamide gel electrophoresis and transferred to nitrocellulose membrane (Amersham, Arlington Heights, USA). Membranes were probed with primary antibodies and the signal was detected using peroxidase-conjugated anti-mouse or anti-rabbit secondary antibodies (Jackson Immunoresearch Labs, Inc., Baltimore, USA). The enhanced chemi-luminescence (ECL) system (Amersham) was used for detection. The following primary Antibodies (Abs) were used: phosphorylated (Thr202/Tyr204) and total ERK1/2, BRAF, phosphorylated (Tyr1173), phosphorylated (Tyr1068) and total EGFR, phosphorylated (Tyr1248) and total HER2, phosphorylated (Ser217/221) and total MEK1/2, phosphorylated pP90^*RSK*^ (Ser380) (from Cell Signaling Technology Inc. Beverly, USA) and CRAF (from Santa Cruz Biotechnology, Santa Cruz, CA). To control the amount of proteins transferred to nitrocellulose membrane, β-actin, Hsp70 and Tubulin were used and detected by anti β-actin mAb (clone AC-15, Sigma, St. Louis, USA), anti Hsp70 mAb (from Calbiochem Merck Biotechnology), and anti-Tubulin mAb (from Abcam, Cambridge, MA, USA). Image detection was performed with Amersham Hyperfilm ECL (Amersham, Amersham, Chicago, IL; Figs. 1, 4, 7; Additional file 1: Fig. S1). For the organoids, protein lysates were fractionated by SDS-PAGE, transferred to a polyvinylidene difluoride (PVDF) membrane and then blocked with 5% BSA in TBST (1% Tween 20, tris-buffered saline). Proteins shown in Fig. 5 were detected on Kodak films (Sigma-Aldrich, St. Louis, MO), using HRP-conjugated secondary antibodies.

### Immunoprecipitation (IP)

For immunoprecipitation cells were rinsed three times with ice-cold PBS, scraped with CHAPS buffer (0.3% CHAPS; 40 mM HEPES; 120 mM NaCl; 1 mM EDTA; 50 mM NaF; 10 mM Glycerol phosphate and 10X pyrophosphate) and lysed by incubation on ice for 30 min after 4 h of treatments. RAF-1 antibody agarose conjugate (Santa Cruz Biotechnology) was used as primary Abs and incubated over night at 4 °C. The immunoprecipitates were collected by centrifugation at 5000 rpm for 5 min at 4 °C and after 2 wash in CHAPS buffer re-suspended in 20 μl of the same buffer and Ladder buffer 4X. The immune complexes were analyzed by Western blot analyses with mouse anti-CRAF antibody. Protein lysates were also subjected to Western blot analyses with BRAF, CRAF and β-actin antibodies. Image detection was performed with Amersham Hyperfilm ECL (Amersham; Fig. 1; Additional file 1: Figure S1).

### Statistical analysis

The Mann-Whitney U test was used for comparing quantitative variables. Results with two-tailed *P* values < 0.05 were judged to be statistically significant. Statistical analysis was performed with SPSS 21.0 software (SPSS, Chicago, IL). Synergism, additivity, and antagonism were assessed by isobologram analysis with a fixed-ratio experimental design using the Chou-Talalay method [27]. Results were analyzed with the Calcusyn software (Biosoft, Cambridge, United Kingdom) and Combination Index (CI) were appropriately derived. By this method, an average CI at the ED50, ED75, and ED90 < 1 indicates synergism, = 1 indicates additivity, and > 1 indicates antagonism, respectively. For organoids analysis, IC_50_ and the CI were calculated according to the Chou-Talalay method using Compusyn. All the experimental methods were performed in accordance with the institutional National and International guidelines and regulations.

## Results

### Selective BRAF inhibition causes paradoxical ERK activation in *BRAF*-wt/*KRAS*-mut contexts

First, we investigated the effects of BRAF and/or MEK inhibition (using dabrafenib and trametinib, respectively) on MAPK pathway activation in *KRAS*-mut lung and pancreatic cancer cell lines (Additional file 1: Table S1). In both A549 (*KRAS*-mut/*BRAF*-wt lung adenocarcinoma) and MiaPaCa2 (*KRAS*-mut/*BRAF*-wt PDAC) exposure to the BRAF-selective inhibitor dabrafenib for 4 h resulted in the dose-dependent phosphorylation of MEK, ERK and p90^*RSK*^, revealing a paradoxical MAPK activation (Fig. 1a); such paradoxical activation was maintained or increased up to 72 h (data not shown). Results in terms of MAPK pathway activation were more variable in response to the pan-RAF inhibitor RAF265; however, paradoxical ERK activation was observed in both cell lines, but only at low/intermediate concentrations (0.1 to 2.5 μM), with relatively little changes occurring at the level of MEK and p90^*RSK*^ phosphorylation (Fig. 1b). Single-agent treatment with the MEK inhibitor trametinib efficiently inhibited ERK phosphorylation (Fig. 1c-d) and strikingly reduced RAF inhibition-induced ERK and p90^*RSK*^ phosphorylation, thereby blunting the paradoxical effect (Fig. 1c-d). Similar results were obtained in all the *KRAS*-mut lung (A427) and pancreatic (PACA44, HPAFII, L3.6pl and PANC1) cancer cell lines analyzed. Paradoxical activation of MEK/ERK signaling following BRAF inhibition has been primarily ascribed to the formation of BRAF/CRAF heterodimers that stimulate ERK signaling [11]. To explore this possibility in our system, we analyzed the formation of BRAF/CRAF complexes in response to either dabrafenib or RAF265. As assessed by IP/WB, both RAF inhibitors caused BRAF/CRAF heterodimer formation in A549 cells (Fig. 1e); similar results were obtained in the *KRAS*-mut pancreatic cancer cell line HPAFII (Additional file 1: Figure S1).

### Functional effects of simultaneous RAF and MEK inhibition in BRAF-wt/RAS-Mut contexts

**Fig. 2:**
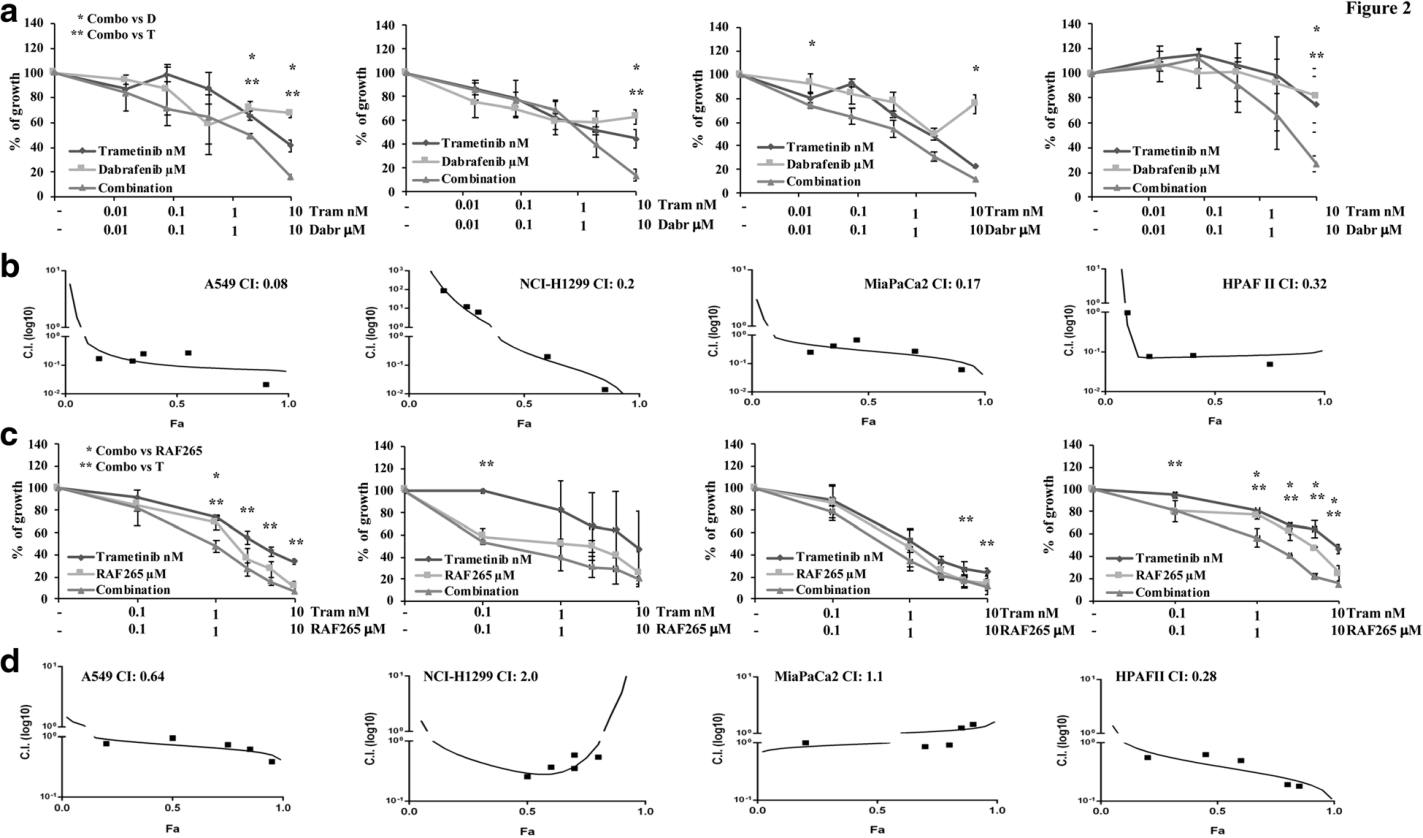
Effects of simultaneous RAF and MEK inhibition in different genetic contexts. **a** and **c** *RAS*-mut lung (A549, *KRAS*-mut, and NCI-H1299, *NRAS*-mut) and pancreatic (MiaPaCa2 and HPAFII, both *KRAS*-mut) cells were exposed to increasing concentrations of trametinib (0.01–10 nM) and either BRAF inhibitor dabrafenib (0.01–10 μM) or the Pan-RAF inhibitor RAF265 (0.1–10 μM); combination experiments were performed using fixed ratio 1:1000. Cells were exposed to treatments for 72 h and cell viability was assessed by Crystal violet assay. The results represent the average ± SD of three independent experiments. **b** and **d** CI were calculated by conservative isobologram analysis for experimental data by this method, an average CI at the ED_50_, ED_75_, and ED_90_ < 1 indicates synergism, =1 indicates additivity, and > 1 indicates antagonism. Isobologram graphs were obtained plotted CI against the fraction affected. Asterisks indicate statistically significant differences (*p* < 0.05 by 2-tailed Student’s t test) for the comparison between * combination- and dabrafenib-treated cells or ** combination- and trametinib-treated cells

We then analyzed the functional effects of combined BRAF and MEK inhibition on in vitro cell growth. In *RAS*-mut lung (A549, *KRAS*-mut, and NCI-H1299, *NRAS*-mut) and pancreatic (MiaPaCa2 and HPAFII, both *KRAS*-mut) cancer cell lines exposed to increasing concentrations of dabrafenib, trametinib, or their combination (at a fixed 1000:1 ratio) for 72 h, simultaneous inhibition of both BRAF and MEK resulted in highly synergistic growth inhibition (with CI ranging from 0.08 to 0.32, Fig. 2a-b and Additional file 1: Table S1); functional synergism was confirmed using different techniques for viability assessment (MTT, Additional file 1: Figure S2A-B; TiterGlow, data not shown) and appeared to be due to decreased proliferation, as shown by greater accumulation of cells in the G1 and lower percentage of cells in the S phases of the cell cycle, in response to combined treatment (Additional file 1: Figure S2C-D). More variable pharmacologic interactions were observed in lung (A549 and NCI-H1299) and pancreatic (MiaPaCa2 and HPAFII) cancer cell lines with the combination of RAF265 and trametinib; indeed, combined treatment was synergistic in A549 and HPAFII (CI: 0.64 and 0.28, respectively) and additive/antagonistic in NCI-H1299 and MiaPaCa2 (CI: 2 and 1.10, respectively) (Fig. 2c-d).

**Fig. 3:**
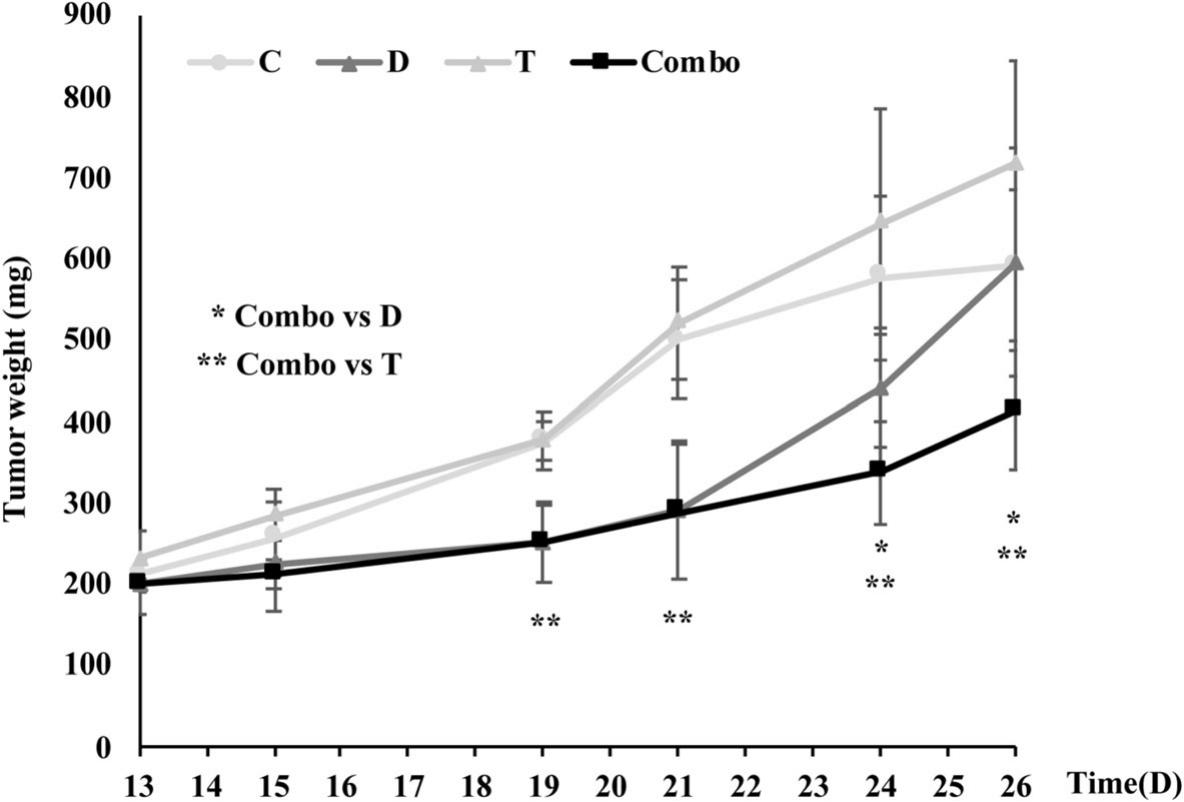
Effects of single and combined MEK and RAF inhibition in *xenograft* models. MiaPaCa2 cell lines were intramuscularly injected into immunodeficient athymic mice. After tumors became palpable (day 13), mice were treated with Vehicle (C), dabrafenib (D), trametinib (T) or combination (Combo) for 2 weeks. The longest perpendicular diameters of each individual tumor was measured at different time points and tumor weight was calculated. Results from one representative experiment out of two independent ones performed are shown. Asterisks indicate statistically significant differences (*p* ≤ 0.02 by 2-tailed Student’s t test) for the comparison between * combination- and dabrafenib-treated mice or ** combination- and trametinib-treated mice

In vitro synergism with combined dabrafenib and trametinib was further confirmed in PDAC xenograft models in vivo, using the MiaPaCa2 cell line; indeed, as shown in Fig. 3, combined dabrafenib and trametinib afforded significantly greater tumor growth inhibition, as compared to either agent alone (p for the comparison between combination and dabrafenib: 0.02 at days 24 and 26 after treatment start; p for the comparison between combination and trametinib: < 0.01 from day 15 onward). We also analyzed the functional effects of combined dabrafenib and trametinib in other lung (*n* = 5) and pancreatic (*n* = 6) cancer cell lines characterized for *KRAS/BRAF* mutational status (Additional file 1: Table S1). Combined treatment resulted in synergistic growth inhibition in 2 out of 3 *KRAS*-mut NSCLC cell lines (Additional file 1: Figure S3) but, was frankly antagonistic in two *KRAS* wild-type cell lines (Additional file 1: Table S1). Similarly, combined dabrafenib and trametinib resulted in growth-inhibitory synergism in 5/6 pancreatic cancer cell lines (all carrying a *KRAS* mutation, with the exception of T3 M4 and the non-neoplastic cell line HPDE) and frank antagonism in the *KRAS*-mut PANC1 cell line (Additional file 1: Table S1 and Additional file 1: Figure S4).

### Effects of combined BRAF/MEK inhibition in patient-derived lung CSC and pancreatic cancer organoids

**Fig. 4:**
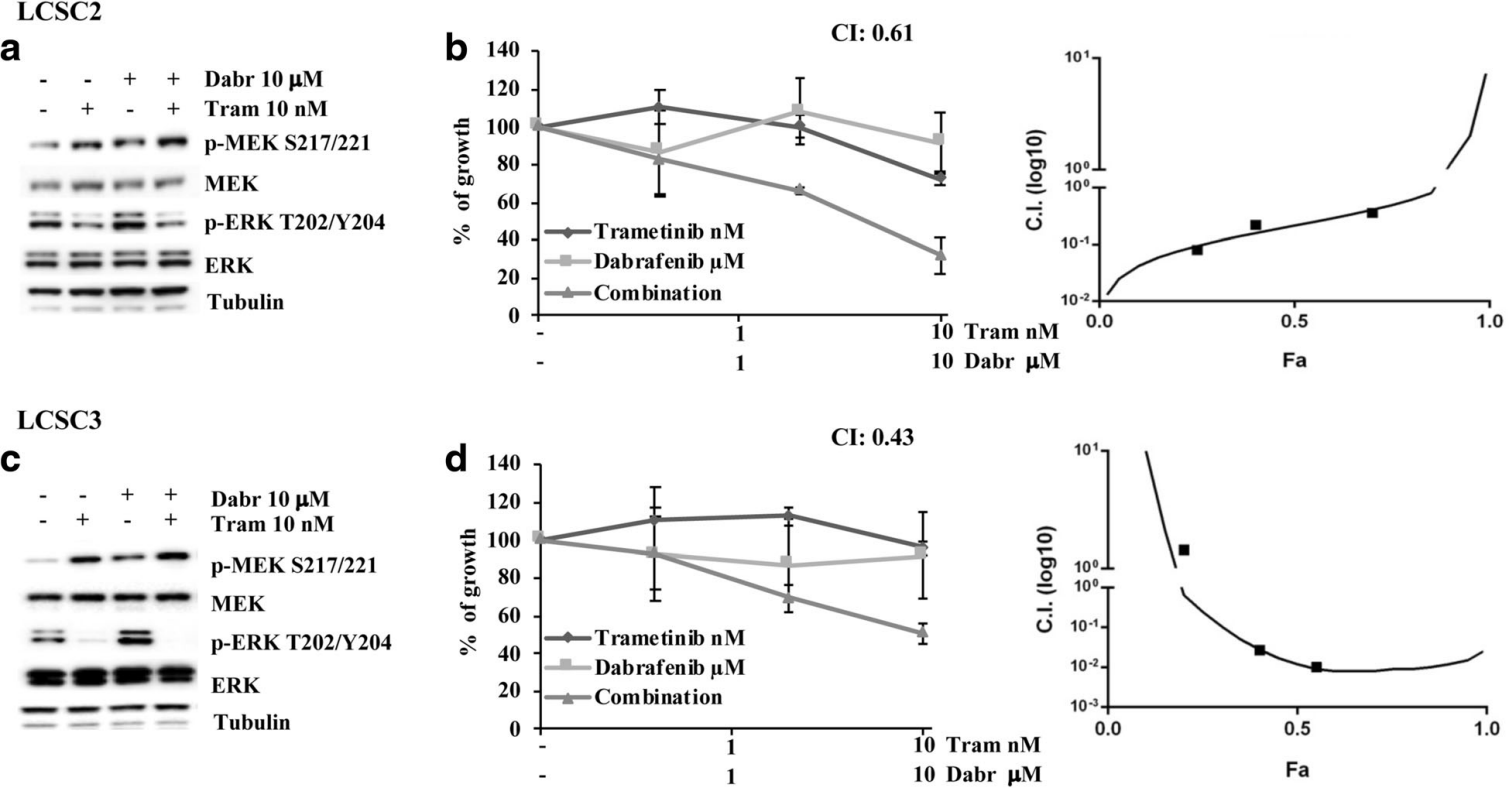
Effects of single and combined MEK and RAF inhibition in LCSC. **a** and **c**. Cells obtained from lung cancer spheres (LCSC) dissociation were treated with trametinib (10 nM) and dabrafenib (10 μM) alone or in combination for 4 h. The cells were lysed and analyzed by Western Blotting using antibodies specific for the protein above indicated. Western blot with Tubulin specific antibody is shown as protein loading and blotting control. **b** and **d**. LCSC2 and LCSC3 cells were plated in 96-well flat-bottom plates; trametinib and dabrafenib were added at their final concentration (1–10 nM), as single agents or in a fixed dose-ratio combination (1:1000). The results represent the average ± SD of three independent experiments. CI were calculated by conservative isobologram analysis for experimental data and plotted against the fraction affected

We next examined the molecular and functional response to single and combined BRAF/MEK inhibition in a panel of 6 LCSC lines (LCSC1, LCSC2, LCSC3, LCSC4, LCSC5, LCSC6) derived from patients with NSCLC of different histological origin (large-cell neuroendocrine carcinoma: LCSC1; squamous cell carcinoma: LCSC2, LCSC3 and LCSC4; adenocarcinoma: LCSC5, LCSC6). As shown in Fig. 4a and c for LCSC2 and LCSC3, selective BRAF inhibition with dabrafenib resulted in paradoxical ERK phosphorylation, while MEK inhibition with trametinib strongly inhibited both basal and BRAF inhibition-induced ERK activation. From a functional point of view, the combination of dabrafenib and trametinib strongly inhibited the in vitro growth of LCSC2 and LCSC3, resulting in a highly synergistic pharmacologic interaction (CI 0.61 and 0.43 in LCSC2 and LCSC3, respectively; Fig. 4b and d). Conversely, in the other LCSC tested single-agent dabrafenib had variable growth-inhibitory effects, single-agent trametinib was relatively ineffective, and their combination did not afford increased growth inhibition (Additional file 1: Table S1 and Figure S5).

**Fig. 5:**
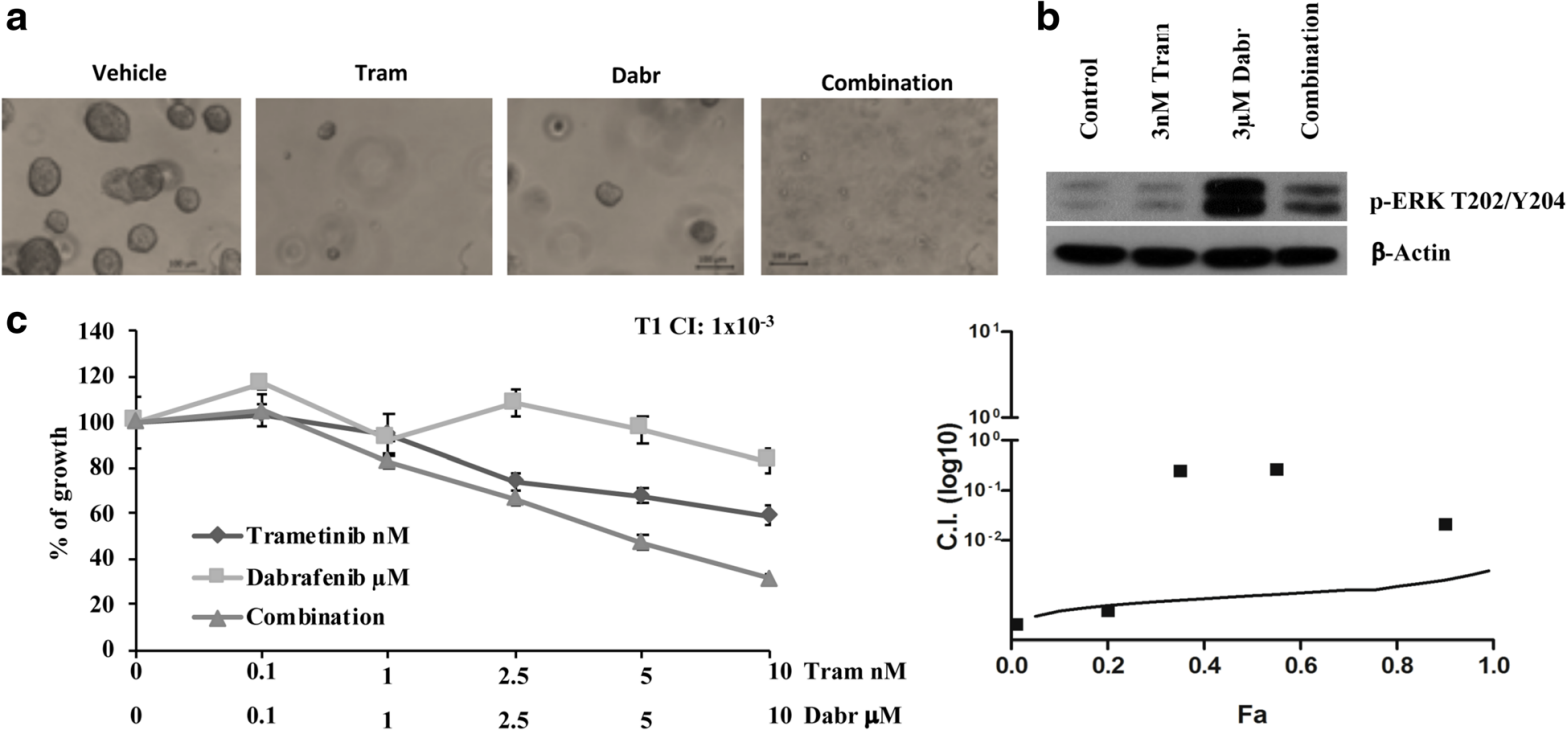
Effects of single and combined MEK and RAF inhibition in patient-derived pancreatic organoids. **a**. Phase-contrast images showing an organoid formation assay. Organoids were seeded as singles cells and treated after 48 h with different doses of trametinib (10 nM,), dabrafenib (10 μM) and combination ratio 1:1000. Images were detected with EVOS Cell Imaging System (**b**). Patient-derived pancreatic organoid T1 (*KRAS*-mut) was incubated with dabrafenib and trametinib alone or in combination for 24 h. Protein lysate were analyzed by Western Blotting using p-ERK antibody. β-Actin is used as protein loading at blotting control. **c**. Patient-derived pancreatic organoid T1 was treated with increasing amounts of the dabrafenib (0.1–10 μM) alone or in combination with trametinib (01–10 nM) ratio 1:1000, for 72 h. Cell viability was evaluated by Cell Titer Glo 2.0 assay. The table shows the CI of trametinib and dabrafenib in a normal pancreatic organoid N1 and in 4 different PDAC organoids *KRAS*-mut

Next, we explored the responses of patient-derived PDAC organoids (Fig. 5a) to combined treatment with dabrafenib and trametinib. We tested 4 neoplastic organoids from individual patient T1, T5, T9 and T14 (all *KRAS*-mut; Additional file 1: Table S1 and Fig. 5); the non-cancer organoid N1 was used as control. As shown in Fig. 5b for the T1 organoid, selective BRAF inhibition with dabrafenib resulted in paradoxical ERK phosphorylation, while trametinib in combination with dabrafenib strongly inhibited BRAF inhibition-induced ERK activation. Strongly synergistic growth inhibitory effects were observed with the combination in 3/4 cancer-derived organoids obtained from pancreatic resections, including T1 (CI: 1 × 10^− 3^; Fig. 5c and Additional file 1: Table S1).

### Efficacy of the combination of trametinib and dabrafenib in isogenic cell lines with different *KRAS* mutational background

**Fig. 6:**
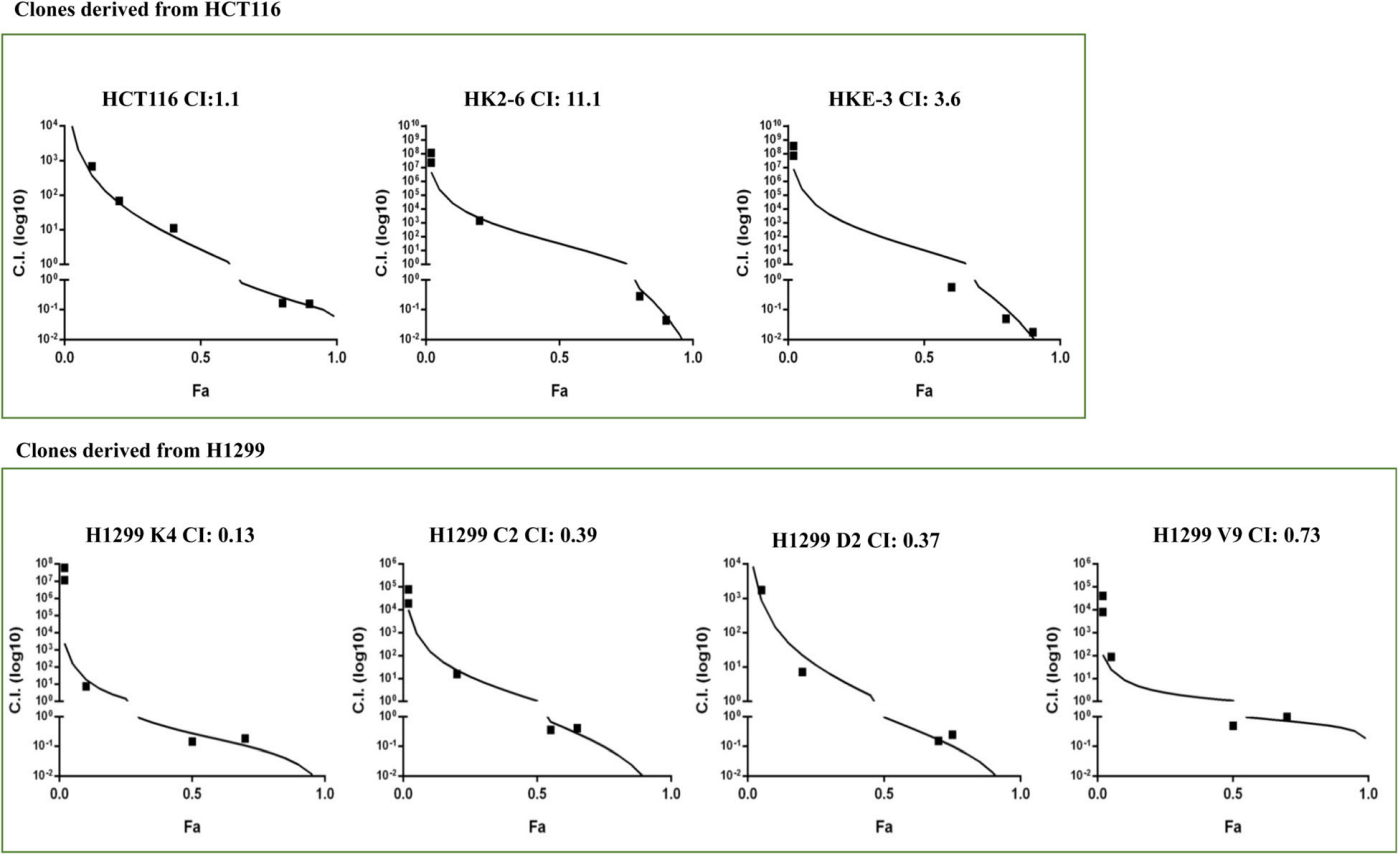
Effects of combined BRAF/MEK inhibition in isogenic cell lines with different *KRAS* mutational background***.*** Growth inhibitory interactions between trametinib and dabrafenib were assessed in CRC cancer cells HCT116 and clones HKE-3 (*KRAS*^*G13D/G13D*^) and HK2–6 (*KRAS*^*wt/wt*^) and in NSCLC cell line H1299 K4 (*KRAS*-wt) and clones C2 (*KRAS*^*G12C*^) D2 (*KRAS*^*G12D*^) and V9 (G12V). Viability was then assessed after 72 h by Crystal violet assay and pharmacologic interactions were evaluated using the Calcusyn software. CI were plotted against the fraction affected

In the entire panel of cell lines tested (Additional file 1: Table S1), the association between *KRAS* mutational status and functional response to combination treatment did not reach statistical significance (*p* value according to Mann-Whitney test 0.84; Additional file 1: Figure S6); however, since the combination appeared to be synergistic in the majority of *KRAS*-mut cell lines and the occurrence of paradoxical ERK activation upon selective BRAF inhibition was originally described in *KRAS*-mut contexts [11], we investigated the effects of dabrafenib and trametinib, alone and in combination, in two *KRAS* isogenic tumor cell line models. In the HCT116 (*KRAS*^*G13D/wt*^) colorectal cancer cell line the combination had additive effects (CI: 1.1), which became frankly antagonistic in both HK2–6 (KRAS^*G13D/G13D*^; CI: 11.1) and HKE-3 (KRAS^*wt/wt*^; CI: 3.6) (Fig. 6). Furthermore, in H1299 (*NRAS*-mut lung adenocarcinoma), in which combined treatment was clearly synergistic (CI: 0.13), enforced expression of different *KRAS* isoforms (G12C, G12D, G12V) did not substantially modify the nature of pharmacologic interactions between dabrafenib and trametinib, which remained synergistic in all *KRAS*-mut clones, although to a slightly lesser extent in H1299 V9 (*KRAS*^*G12V*^, CI: 0.73, Fig. 6). Overall, these results support the hypothesis that *RAS* mutational status is not the sole determinant of functional synergism between RAF and MEK inhibitors.

### Potential role of RTKs in paradoxical MAPK activation

**Fig. 7:**
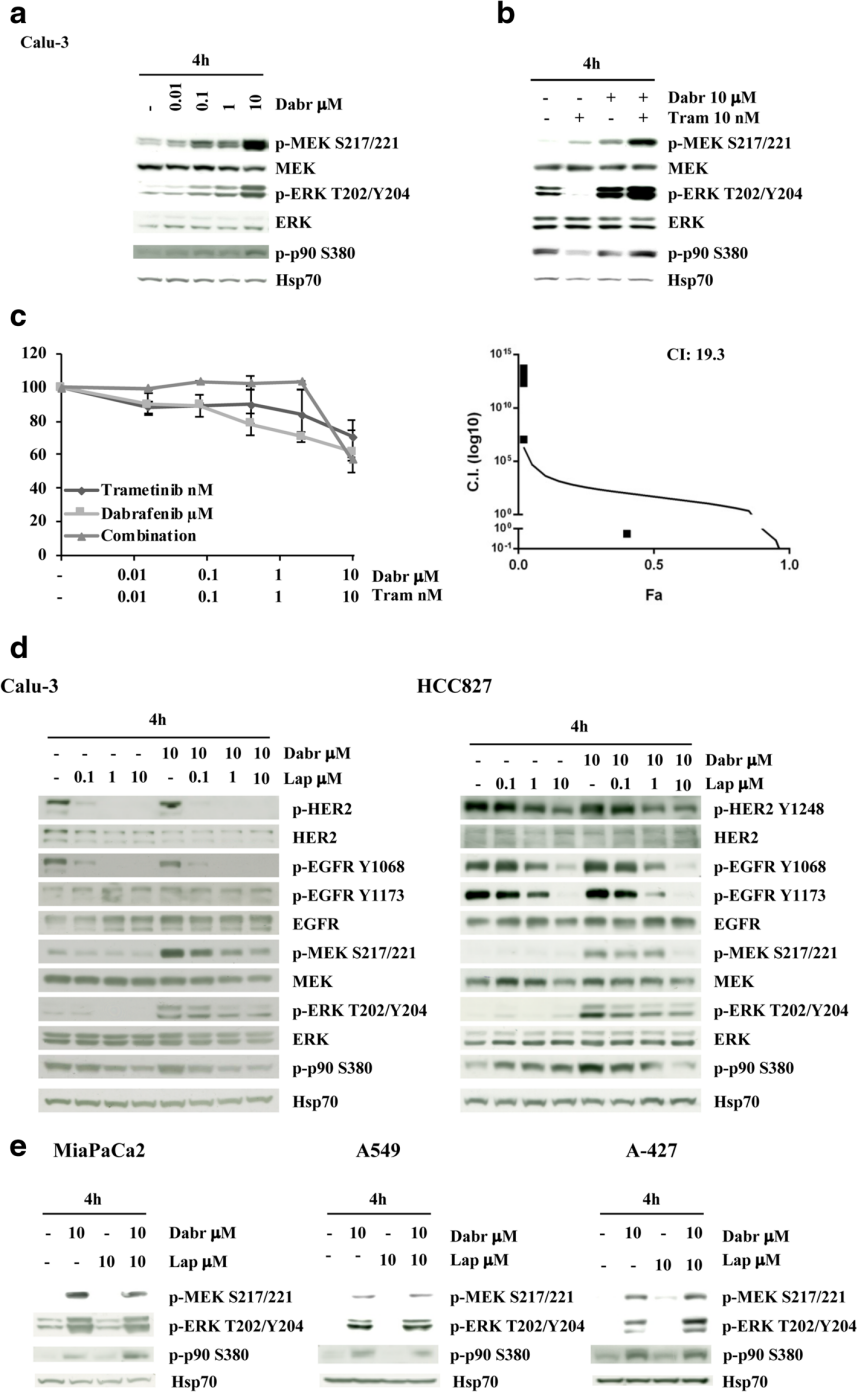
Selective BRAF inhibition induces EGFR family-dependent MAPK hyperactivation in Calu-3 cells. **a** and **b** The NSCLC cell line Calu-3 (*HER2*-amplified, *KRAS*-wt) was treated with increasing concentrations of dabrafenib (0.01–10 μM) alone **a** or in combination with trametinib (ratio 1:1000; **b**) for 4 h. The cells were lysed and analyzed by Western Blotting using antibodies specific for the proteins indicated. Western blot with Hsp70 specific antibody is shown as protein loading and blotting control. **c** Calu-3 cells were treated with increasing concentrations of dabrafenib (0.01–10 μM) and trametinib (0.01 nM–10 nM) alone or in combination for 72 h. Cell viability was assessed by Crystal violet assay and pharmacologic interactions were evaluated using the Calcusyn software. The results represent the average ± SD of three independent experiments. **d** Calu-3 and HCC827 (*EGFR*-mut) cells were treated with increasing concentrations of dabrafenib (0.1–10 μM) and lapatinib (0.1–10 μM) for 4 h. The proteins were subjected to Western Blotting and analyzed for the indicated antibodies. **e** MiaPaCa2, A549 and A427 cells were treated with dabrafenib (10 μM) and lapatinib (10 μM) alone or in combination for 4 h. The cells were lysed and analyzed by Western Blotting using antibodies specific for the protein above indicated

In Calu-3 (HER2-amplified, *KRAS*-wt, lung squamous cell carcinoma), dabrafenib-induced paradoxical ERK activation could not be abrogated by simultaneous MEK inhibition (Fig. 7a-b) and pharmacological interactions between the two drugs were highly antagonistic (CI: 19.3; Fig. 7c). We thus investigated whether EGFR family members may be involved in paradoxical MAPK activation in *RAS*-wt, EGFR family-driven contexts. In Calu-3 (HER22-amplified) and HCC827 (*EGFR*-mut), in which both EGFR and HER2 are constitutively active, selective BRAF inhibition by dabrafenib induced a strong paradoxical activation of the MAPK cascade (p-MEK, p-ERK, and p-p90^*RSK*^) (Fig. 7d). In these contexts, MAPK activation was found to be dependent on EGFR family activation: indeed, the EGFR/HER2 inhibitor lapatinib strongly reduced dabrafenib-induced MAPK activation (Fig. 7d). Conversely, in *KRAS*-mut contexts (MiaPaCa2 pancreatic cancer; A549 and A-427 NSCLC), EGFR family activation was dispensable for dabrafenib-induced paradoxical ERK activation, as the addition of lapatinib to dabrafenib had no appreciable effect on ERK activation (Fig. 7e). From a functional point of view, in EGFR-dependent NSCLC models (HER2-amplified, Calu-3; *EGFR*-mut, HCC827 and NCI-H1650) double EGFR/HER2 blockade by lapatinib resulted in striking growth inhibitory effects in vitro, which were not substantially increased by simultaneous exposure to MAPK pathway inhibitors (either dabrafenib or trametinib, Additional file 1: Figure S7).

## Discussion

In this study, we investigated the potential of a combinatorial treatment with small molecules targeting key components of the MAPK pathway, namely BRAF and MEK, in *KRAS*-mut NSCLC and PDAC. In line with current literature, selective BRAF blockade resulted in paradoxical downstream MAPK activation, which was reversed by simultaneous MEK inhibition. In a consistent proportion (21/32, 65%) of the models examined (both cell lines and patient-derived lung CSC and PDAC organoids), the “vertical” combination of dabrafenib and trametinib (targeting BRAF and MEK) resulted in synergistic tumor growth inhibition in vitro, mostly due to greater inhibition of proliferation achieved with combined treatment. In the MiaPaCa2 xenograft model, combined dabrafenib and trametinib also resulted in significantly greater tumor growth inhibition in vivo, as compared to each agent alone. Pan-RAF inhibitors (such as RAF265) had more variable effects in terms of both paradoxical MAPK activation and functional synergism with MEK inhibitors. Although the combination appeared to be synergistic in the majority of *KRAS*-mut cell lines, no definitive mechanistic association between *KRAS* mutational status and functional synergism with combined BRAF/MEK inhibition was found, supporting the idea that *RAS* mutational status is not the sole determinant of functional synergism between RAF and MEK inhibitors. Indeed, in *KRAS*-wt contexts, paradoxical MAPK activation in response to BRAF inhibition may still occur and appears to be mediated by upstream signaling through RTKs (namely EGFR family members).

The majority of PDAC (~ 90%) and a high proportion (25–40%) of NSCLC harbor oncogenic activation of *KRAS* [28–31]*,* which have been demonstrated to be necessary to both initiate and maintain tumorigenesis [32]. Mutant *KRAS* engages several downstream pathways, including the MAPK signaling cascade, to execute its oncogenic program [28]. While *RAS* mutations are an established resistance factor to an array of targeted agents [33, 34], clinical attempts at targeting RAS activity, either directly or by targeting common downstream effectors, have failed so far [35]. One appealing strategy would be to target MAPK activation downstream of RAS: indeed, RAF proteins have been shown to be indispensable for RAS-dependent transformation and progression in several cancer models [36–38]; moreover, early evidence has suggested that the presence of *KRAS* mutations might portend sensitivity to MEK inhibitors [39–45]. However, clinical translation of single-step RAF or MEK inhibition in cancer has been hampered by complex feedback mechanisms that are not only able to reactivate targeted pathways following treatment [18, 46], but also specifically and differentially operate in different tumor genetic backgrounds. According to currently accepted mechanistic models, we show here that exposure of *KRAS*-mutant cells (including lung CSC and PDAC organoids) to BRAF-selective inhibitors results in the paradoxical activation of the MEK/ERK module, due to the formation of CRAF/BRAF dimers, as previously reported [10–12]. Conversely, in BRAF^V600E^ melanomas no BRAF/CRAF heterodimer formation was observed and both BRAF- and MEK-selective inhibitors efficiently shut down ERK activation (Cesta Incani et al., 2018, manuscript in preparation), even when used as single agents. As a consequence, simultaneous RAF/MEK inhibition has a strong mechanistic rationale in *BRAF*-wt (and particularly in *KRAS*-mut) genetic contexts [18–20]. Our findings do support this concept, as striking growth-inhibitory synergism was observed with dabrafenib and trametinib in the majority of NSCLC and PDAC models tested (both cell lines and, most importantly, patient-derived lung CSC and PDAC organoids). However, a few important points remain to be clarified: 1) whether selective or broad-spectrum RAF-isoform inhibition might be more advantageous in combination with MEK inhibitors; 2) the role of *KRAS*-mut as a potential selection biomarker for the “vertical” RAF/MEK inhibitor combination.

Literature data suggest that CRAF inhibition is crucial to enable MEK inhibitors to effectively inhibit downstream signaling to ERK1/2 [18] and that pan-RAF inhibitors could more potently synergize with MEK inhibitors, as compared with BRAF-selective inhibitors [20]. In our hands, the pan-RAF inhibitor RAF265 had more variable effects, as compared to the BRAF-selective inhibitor dabrafenib, on both paradoxical ERK activation and growth-inhibitory synergism; such discrepancies may be due to several different reasons, including the different dynamics and dose-dependency of inhibition of individual RAF isoforms, cellular ATP levels [10, 47], the specific genetic background of the models tested, and the specific site of action of the MEK inhibitors being combined [18]. Overall, while the general strategy of inhibiting both RAF and MEK along the MAPK cascade appears to be promising even outside the clinically validated *BRAF*-mut genetic context (see also earlier work conducted by our group in leukemia models, [48]), the optimal selection of agents/mechanisms of action of RAF and MEK inhibitors to be combined remains to be addressed, taking into account the clinical tolerability profile of individual agents/combinations.

Paradoxical MAPK pathway activation in response to BRAF-selective inhibitors was initially closely linked to *RAS* mutational status, in fact this effect was absent in cells with wild-type RAS, while it was restored after the introduction of an oncogenic RAS [49, 50]. Together with evidence that “vertical” RAF/MEK blockade was synthetically lethal in *KRAS*-mut contexts [19], such background prompted us to select NSCLC and PDAC, two diseases where *KRAS* mutations are frequent and bear important clinical consequences, as the main target of our analysis. However, synergism analysis across the entire set of cellular models analyzed displayed no significant correlation between *RAS* mutational status and synergistic interactions between dabrafenib and trametinib. Such observation was mechanistically corroborated by evidence that pharmacological interactions between BRAF and MEK inhibitors were not substantially modified in isogenic cellular models of lung and colorectal cancer differing for *KRAS* status. This observation is consistent with evidence that paradoxical MAPK activation in response to BRAF inhibition also occurs in *KRAS*-wt/*BRAF*-wt genetic contexts (as shown in this report and in [49]) and supports the idea that *RAS* mutational status is not the sole determinant of combined treatment outcome. In that respect, complexities in evaluating the role of *RAS* mutations in driving different tumor phenotypes are highlighted by recent evidence showing that distinct evolutionary routes, licensed by defined allelic states and/or combinations of hallmark tumor suppressor alterations (*Cdkn2a*, *Trp53*, Tgfβ-pathway), direct variations of oncogenic RAS dosage gain, to drive the early progression of PDAC and shape its downstream biology [51].

RTKs (and EGFR family members in particular) are a likely candidate to mediate paradoxical MAPK activation in response to inhibition of a single step of the cascade; indeed, EGFR family-mediated (re)activation of either the MAPK pathway itself or other crosstalking pathways (such as the PI3K/AKT axis) has been described in several cancer models, with or without and underlying *BRAF* or *KRAS* mutation [52, 53]. Consistent with these data, here we show that in tumor cellular contexts that are highly dependent on EGFR family signaling, such as the *HER2*-amplified (Calu3) and *EGFR*-mutant (HCC827) NSCLC cell lines, BRAF inhibition paradoxically reactivated the MAPK pathway in an EGFR/HER2-dependent manner; interestingly, we observed a similar EGFR family feedback activation in response to either BRAF or MEK inhibition in colorectal cancer cell lines and patient-derived cancer stem cells [52] (Bazzichetto C., unpublished results). In this specific context, EGFR/HER2 inhibition by lapatinib was able to shut down paradoxical MAPK hyperactivation in response to dabrafenib, whereas in *KRAS*-mutant cell lines, in which the need for EGFR family activation is bypassed by constitutively active RAS, lapatinib was ineffective in that respect. Based on this evidence, co-targeting of EGFR family members upstream and MAPK signal transducers (RAF, MEK) downstream has been proposed as a promising therapeutic strategy [52, 54, 55] and, in part, validated clinically in *BRAF*-mut CRC [45, 56, 57]. However, we did not observe growth-inhibitory synergism in vitro with combined lapatinib and either dabrafenib or trametinib in *HER2*-amplified or *EGFR*-mutant NSCLC models. This might be due to the high intrinsic sensitivity of these models to lapatinib: accordingly, recent data show that, in colorectal cancer models, combined blockade of EGFR and MEK intercepts heterogeneous mechanisms of acquired resistance to anti-EGFR therapies and results in therapeutic synergism only once resistance to anti-EGFR agents has ensued [54, 58], suggesting that in highly sensitive models the right setting to apply combinatorial strategies might be treatment-resistant disease.

## Conclusions

**Fig. 8:**
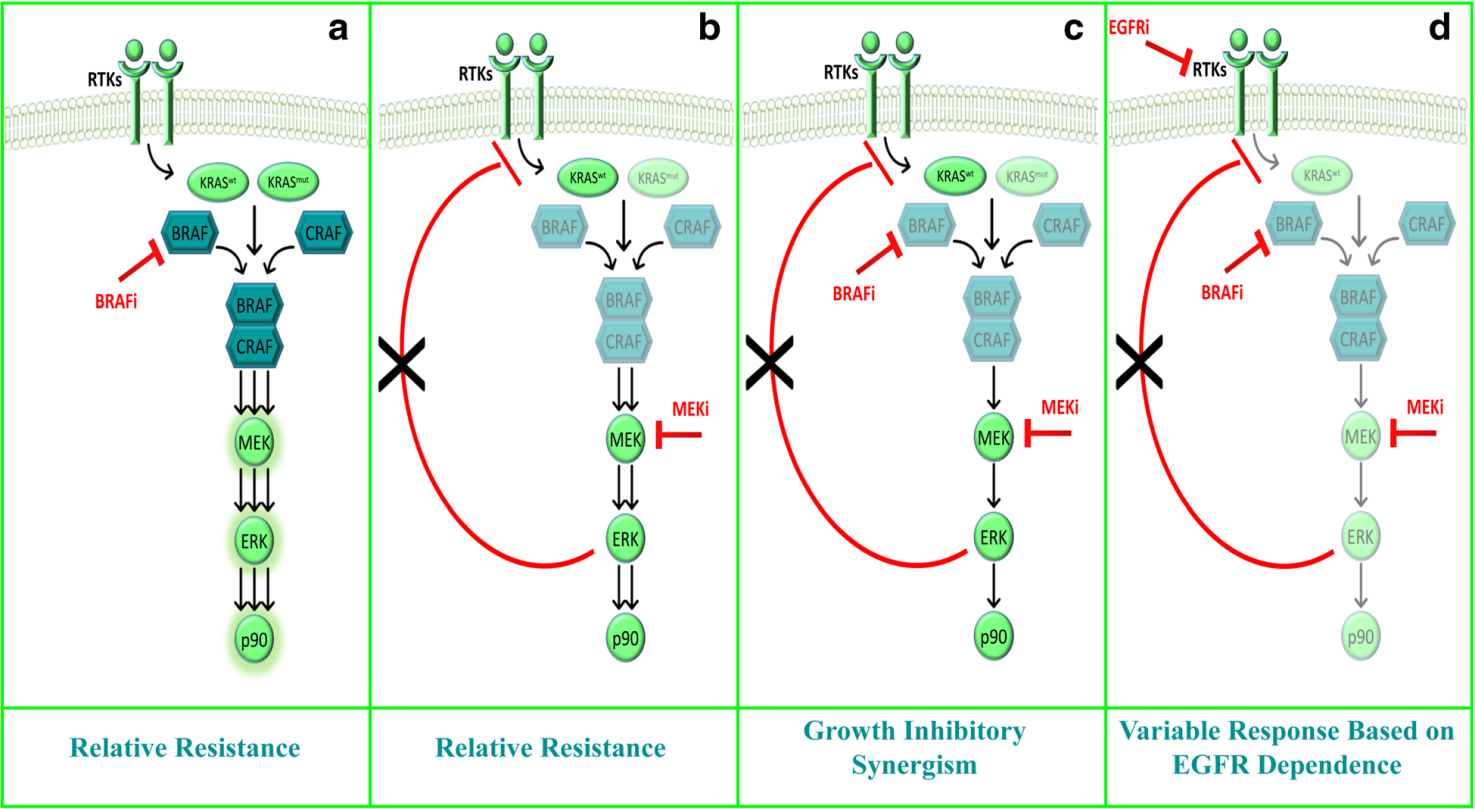
Working model of intra-pathway feedbacks and BRAF/MEK growth-inhibitory synergism. **a** In BRAF-wt/KRAS-mut contexts, selective BRAF inhibition induces BRAF-CRAF dimerization, which hyperactivates the MAPK pathway, thus resulting in relative resistance to treatment. In BRAF-wt/KRAS-wt contexts, paradoxical MAPK activation may be sustained by the RAS-dependent upstream signaling of RTKs (in particular EGFR family members). **b** Upon allosteric MEK inhibition, the MAPK pathway downstream of a mutant KRAS is efficiently shut down; however, MEK inhibition-induced removal of ERK-mediated feedback RTK inhibition may result in incomplete MAPK pathway inhibition or pathway reactivation, again resulting in relative resistance to the drug. **c** Combined BRAF/MEK inhibition results in efficient pathway blockade and a synergistic effect on cell growth inhibition, particularly downstream of a mutant KRAS; however, as highlighted also in panel b, in KRAS-wt contexts removal of ERK-mediated feedback RTK inhibition may result in RTK-dependent pathway (re)activation, thus resulting in only partial blockade of downstream signaling. **d** Thus, in KRAS-wt contexts, triple RTK (EGFR family in the specific case discussed here)/BRAF/MEK inhibition is hypothesized to completely prevent paradoxical MAPK activation; functional growth-inhibitory synergism will then vary according to the degree of intrinsic sensitivity/resistance to RTK inhibition

In conclusion, in this study we show that a “vertical” combination strategy simultaneously targeting BRAF and MEK shuts down BRAF inhibitor-induced paradoxical MAPK activation and may result in therapeutic synergism in preclinical models (both cancer cell lines and patient derived CSC and organoids) of *RAS*-mut NSCLC and PDAC in vitro*.* However, as highlighted in Fig. 8, which recapitulates our data in a coherent working model, the genetic/molecular background of the cancer cell being targeted crucially determines the outcome of drug interactions: indeed, depending on *KRAS* status and EGFR family dependence, combined BRAF/MEK inhibition may be sufficient to prevent paradoxical MAPK activation and afford synergistic growth inhibition or additional EGFR blockade maybe required to completely shut down the pathway and cell growth/survival. Further studies are needed, particularly to identify potential biomarkers to select out patients at highest chance of benefiting from such a promising combinatorial strategy.

## Additional file

Additional file 1: Supplementary Methods. **Figure S1.** RAF inhibition induces BRAF/CRAF heterodimerization in *KRAS*-mut contexts. **Figure S2.** Effects of trametinib, dabrafenib and their combination on cell growth and cell cycle distribution in A549 and MiaPaCa2 cells. **Figure S3.** Effects of trametinib and dabrafenib combination in lung cancer cells. **Figure S4.** Effects of trametinib and dabrafenib combination in pancreatic cancer cells. **Figure S5.** Effects of trametinib, dabrafenib and their combination in LCSC. **Figure S6.** Statistical correlation between *KRAS* status and pharmacological interactions. **Figure S7.** Effects of lapatinib in *EGFR/HER2* amplified lung cancer cell lines. **Table S1*.*** Summary of the genetic status of the cell lines analyzed and response to treatments. (PDF 2519 kb)

## Abbreviations

Ab: Antibody
AKT: Protein kinase B
ATCC: American Type Culture Collection
CI: Combination Index
EGFR: Epidermal Growth Factor Receptor
ERK: Extracellular-signal-Regulated Kinase
HER2: Human Epidermal Growth Factor Receptor 2
IC_50_: 50% of Cell growth Inhibition
IP: Immunoprecipitation
LCSC: Lung Cancer Stem Cells
MAPK: Mitogen-Activated Protein Kinase
MEK: Mitogen-activated protein kinase kinase
mTOR: mammalian Target of Rapamycin
NSCLC: Non-Small Cell Lung Cancer
PDAC: Pancreatic Ductal AdenoCarcinoma
PI3K: PhosphoInositide3-Kinase
RAF: Rapidly Accelerated Fibrosarcoma
RTK: Receptor Tyrosine Kinase
WB: Western Blot
