# Supplementary material for: Therapeutic potential of combined BRAF/MEK blockade in BRAF-wild type preclinical tumor models
Source: J Exp Clin Cancer Res. 2018 Jul 9;37:140. doi: 10.1186/s13046-018-0820-5 (PMC6038340; doi:10.1186/s13046-018-0820-5)
Supplement: Supplementary file 1 — Supplementary Methods. Figure S1. RAF inhibition induces BRAF/CRAF heterodimerization in KRAS-mut contexts. Figure S2. Effects of trametinib, dabrafenib and their combination on cell growth and cell cycle distribution in A549 and MiaPaCa2 cells. Figure S3. Effects of trametinib and dabrafenib combination in lung cancer cells. Figure S4. Effects of trametinib and dabrafenib combination in pancreatic cancer cells. Figure S5. Effects of trametinib, dabrafenib and their combination in LCSC. Figure S6. Statistical correlation between KRAS status and pharmacological interactions. Figure S7. Effects of lapatinib in EGFR/HER2 amplified lung cancer cell lines. Table S1. Summary of the genetic status of the cell lines analyzed and response to treatments. (PDF 2519 kb) [file 13046_2018_820_MOESM1_ESM.pdf]

Title: Therapeutic potential of combined BRAF/MEK blockade in *BRAF*-wild type preclinical tumor models.

Anais del Curatolo[1,2§], Fabiana Conciatori[1,3§], Ursula Cesta Incani[1], Chiara Bazzichetto[1,3], Italia Falcone[1], Vincenzo Corbo[2], Sabrina D'Agosto[2], Adriana Eramo[4], Giovanni Sette[4], Isabella Sperduti[5], Teresa De Luca[6], Mirko Marabese[7], Senji Shirasawa[8], Ruggero De Maria[9], Aldo Scarpa[2], Massimo Broggini[7], Donatella Del Bufalo[6], Francesco Cognetti[1], Michele Milella[1§] and Ludovica Ciuffreda[1§].*

(*E-mail: michele.milella@ifo.gov.it; michelemilella@hotmail.com)

[1] Medical Oncology 1, IRCCS Regina Elena National Cancer Institute, Rome, Italy; anais1@hotmail.it (A.D.C.); fabiana.conciatori@ifo.gov.it (F.C.); ursulacestaincani@virgilio.it (U.C.I); chiara.bazzichetto@ifo.gov.it (C.B.); italia.falcone@ifo.gov.it (I.F.); francesco.cognetti@ifo.gov.it (F.C.); michele.milella@ifo.gov.it (M.M.); ludovica.ciuffreda@ifo.gov.it (L.C.);

[2] ARC-Net Research Centre and Department of Pathology, University of Verona, Verona, Italy; vincenzocorbo@gmail.com (V.C.); sabrinaluigia.dagosto@univr.it (S.D.A.); aldo.scarpa@univr.it (A.S.);

[3] University of Rome "La Sapienza", Rome, Italy;

[4] Department of Hematology, Oncology and Molecular Medicine, Istituto Superiore di Sanità, Rome, Italy; adriana.eramo@iss.it (A.E.); giovanni.sette@gmail.com (G.S.);

[5] Biostatistics, IRCCS Regina Elena National Cancer Institute, Rome, Italy; isabella.sperduti@ifo.gov.it (I.S.);

[6] Preclinical Models and New Therapeutic Agents Unit, IRCCS Regina Elena National Cancer Institute, Rome, Italy; deluca_teresa@libero.it (T.D.L.); donatella.delbufalo@ifo.gov.it (D.D.B.);

[7] Laboratory of Molecular Pharmacology, Department of Oncology, IRCCS - Istituto di Ricerche Farmacologiche "Mario Negri", Milan, Italy; mirko.marabese@marionegri.it (M.M.);

[8] Central Research Institute for Advanced Molecular Medicine, Fukuoka University, Fukuoka, Japan;

sshirasa@fukuoka-u.ac.jp (S.S.);

[9] Institute of General Pathology, Catholic University of the Sacred Heart, Rome, Italy; demariaruggero@gmail.com (R.D.M.).

§ These authors contributed equally to this work.

Supplementary file Summary

- Supplementary Methods
- Supplementary Figure Legends
- Supplementary Table and Figures

Supplementary Methods

3-[4,5-diMethylThiazol- 2-yl]-2,5-diphenylTetrazolium bromide (MTT) Assay

For MTT (Sigma- Aldrich, St. Louis, MO, USA) assay, tumor cells were dispensed into 96-well (Corning, Inc.) at variable concentration (1000 cells/well for A549 and 5000 cells/well for MiaPaCa2). The following day drugs were added at indicated concentrations. After 72 hours of treatment the MTT made up in medium to a final concentration of 0.5 mg/mL and incubate for 4 hours at 37°C. Subsequently the MTT was removed and 100 µl of Isopropanol was then added into each well to dissolve purple formazan crystals. After for 30 minutes of incubation the absorbance was measured at 570 nm.

Flow cytometric analysis of cell cycle

The cell cycle analysis was performed on A549 and MiaPaCa2 cell lines treated with trametinib or dabrafenib alone or in combination for 48 hours. After treatment, the cells were trypsinized and fixed in cold 70% ethanol overnight. The cells were washed with PBS and then stained with Propidium Iodide (PI) solution (1 µg/µL PI and 0.125% RNaseA; Sigma Aldrich, St. Louis, MO) at room temperature. Approximately 10.000 cells/sample were analyzed using BD FACSCalibur flow cytometer.

Supplementary Figure Legends

Figure S1. RAF inhibition induces BRAF/CRAF heterodimerization in *KRAS*-mut contexts. HPAFII cells were treated with trametinib (10nM), dabrafenib (10µM) or RAF265 (10µM) for 4 hours. Endogenous CRAF was immunoprecipitated and the immunocomplex was blotted for CRAF. In total cell lysates, BRAF and CRAF levels were shown.

Figure S2. Effects of trametinib, dabrafenib and their combination on cell growth and cell cycle distribution in A549 and MiaPaCa2 cells. Cells were exposed to increasing concentration of trametinib (0.1-10 nM) and dabrafenib (0.1-10 µM) alone or in combination using a using a fixed-ratio 1:1000 and then assessed for cell viability (by MTT assay) and cell cycle distribution (by PI staining). **A and B.** CI were calculated by conservative isobologram analysis and plotted against the fraction affected. **C and D.** Histograms represent DNA contents of cell cycle phases G0/G1phase , S phase and G2/M phase. Results from one experiment representative of three are shown.

Figure S3. Effects of trametinib and dabrafenib combination in lung cancer cells. Lung cancer cells were treated with the combination of trametinib (0.01-10 nM) and dabrafenib (0.01-10 µM), at

fixed ratio (1:1000) for 72h and cell viability was assessed by Crystal violet assay. CI were calculated by conservative isobologram analysis for experimental data and plotted against the fraction affected.

Figure S4. Effects of trametinib and dabrafenib combination in pancreatic cancer cells. Pancreatic cancer cells were treated with the combination of trametinib (0.01-10 nM) and dabrafenib (0.01-10 μM), at fixed ratio of doses (1:1000) for 72h. CI were calculated by conservative isobologram analysis for experimental data and plotted against the fraction affected.

Figure S5. **Effects of trametinib, dabrafenib and their combination in LCSC.** LCSC1, LCSC4, LCSC5 and LCSC6 cell lines were treated with trametinib and dabrafenib, alone or in combination, using a fixed dose ratio (1:1000). Cell viability was assessed by Crystal violet assay after 72 h. Results Are expressed as percentage of growth inhibition relative to untreated control and represent the average ± SEM of three independent experiments. Pharmacologic interactions were evaluated using the Calcusyn software.

Figure S6. Statistical correlation between *KRAS* status and pharmacological interactions. Box plot shows the relationships between trametinib/dabrafenib pharmacologic interactions (CI) and KRAS status in the panel of analyzed cell lines.

Figure S7. Effects of lapatinib in *EGFR/HER2* amplified lung cancer cell lines. Lung cancer cells were treated with the combination of trametinib and lapatinib, alone or in combination (left panels) or dabrafenib and lapatinib, alone or in combination (right panels) 26 at fixed ratio of doses (1:1000 and 27 1:1, respectively) for 72h. Pharmacologic interactions were evaluated using the Calcusyn software.

Table S1. **Summary of the genetic status of the cell lines analyzed and response to treatments.**

Cell line	*KRAS*	*BRAF*	CI tram/dabr
**Lung**			
A-427	G12D/wt*	wt*	0.70
A549	G12S/G12S*	wt*	0.08
Calu-1	G12C/wt*	wt*	$2.2x10^{4}$
Calu-3	wt/wt*	wt*	19.3
HCC827	wt/wt	wt	0.66
NCI-H460	Q61H/Q61H*	wt*	0.80
NCI-H1299	wt/wt*	wt*	0.2
H1299 (K4)	wt [1]	wt	0.13
H1299 (D2)	G12D [1]	wt	0.37
H1299 (C2)	G12C [1]	wt	0.39
H1299 (V9)	G12V [1]	wt	0.73
**Lung Cancer Stem Cells**			
LCSC1	wt	wt	0.02
LCSC2	wt	wt	0.61
LCSC3	wt	wt	0.43
LCSC4	G12C	wt	316
LCSC5	wt	wt	3
**Pancreas**			
HPDE	wt	wt	0.83
MiaPaCa2	G12C/G12C*	wt*	0.17
T3M4	wt/wt [2]	wt	0.50
PANC1	G12D†	wt	3.90
PACA44	G12V [2]	wt	0.81
HPAFII	G12D/wt †	wt	0.32
PT45P1	G13D†	wt	0.60
L3.6pl	G12D [3]	wt	0.40
**Organoids**			
N1	wt/wt §	wt	$>1x10^{20}$
T1	G12V/wt§	wt	$1x10^{-3}$
T5	G12R/ wt§	n.a.	$>1x10^{10}$
T9	G12V/wt§	wt	$1x10^{-4}$
T14	G12D/wt§	wt	0.06
**Colon**			
HCT116	G13D/wt* [4]	wt*	1.1
HK2-6	G13D [4]	wt	11.1
HKE-3	wt [4]	wt	3.6

*BRAF and KRAS gene status was assessed by Sanger sequencing (see Supplemetary M&M and Milella M. Sci Rep. 2017).

§ BRAF and KRAS gene status was assessed by Sanger sequencing (https://www.ncbi.nlm.nih.gov/pmc/articles/PMC4334572/#SD15)

† COSMIC website http://www.sanger.ac.uk/cosmic/

1. Garassino MC, Marabese M, Rusconi P, Rulli E, Martelli O, Farina G, Scanni A, Broggini M: **Different types of K-Ras mutations could affect drug sensitivity and tumour behaviour in non-small-cell lung cancer.** *Ann Oncol* 2011, **22:**235-237.
2. Moore PS, Sipos B, Orlandini S, Sorio C, Real FX, Lemoine NR, Gress T, Bassi C, Kloppel G, Kalthoff H, et al: **Genetic profile of 22 pancreatic carcinoma cell lines. Analysis of K-ras, p53, p16 and DPC4/Smad4.** *Virchows Arch* 2001, **439:**798-802.
3. Gysin S, Rickert P, Kastury K, McMahon M: **Analysis of genomic DNA alterations and mRNA expression patterns in a panel of human pancreatic cancer cell lines.** *Genes Chromosomes Cancer* 2005, **44:**37-51.
4. Shirasawa S, Furuse M, Yokoyama N, Sasazuki T: **Altered growth of human colon cancer cell lines disrupted at activated Ki-ras.** *Science* 1993, **260:**85-88.

Figure S1

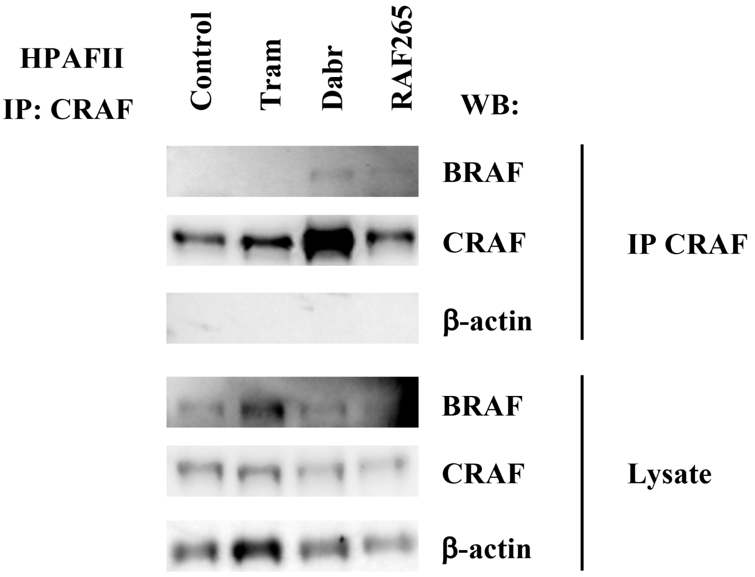
HPAFII
IP: CRAF
Control
Tram
Dabr
RAF265
WB:
BRAF
CRAF
β-actin
IP CRAF
BRAF
CRAF
β-actin
Lysate

Figure S2

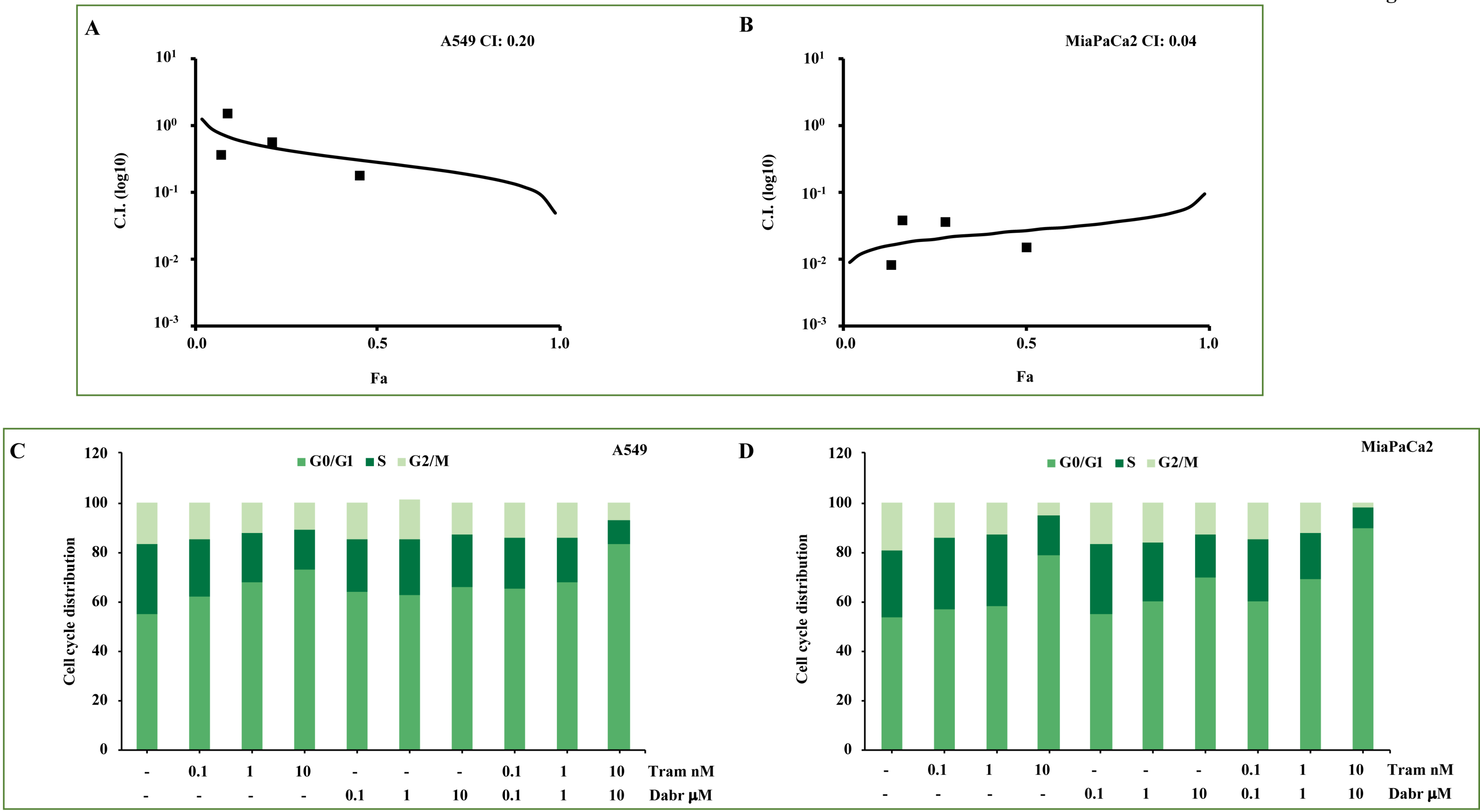
A
A549 CI: 0.20
C.I. (log10)
10^1
10^0
10^-1
10^-2
10^-3
0.0
0.5
1.0
Fa
B
MiaPaCa2 CI: 0.04
C.I. (log10)
10^1
10^0
10^-1
10^-2
10^-3
0.0
0.5
1.0
Fa
C
A549
G0/G1
S
G2/M
Cell cycle distribution
0
20
40
60
80
100
120
Tram nM: - 0.1 1 10 - - - 0.1 1 10
Dabr µM: - - - - 0.1 1 10 0.1 1 10
D
MiaPaCa2
G0/G1
S
G2/M
Cell cycle distribution
0
20
40
60
80
100
120
Tram nM: - 0.1 1 10 - - - 0.1 1 10
Dabr µM: - - - - 0.1 1 10 0.1 1 10

Figure S3

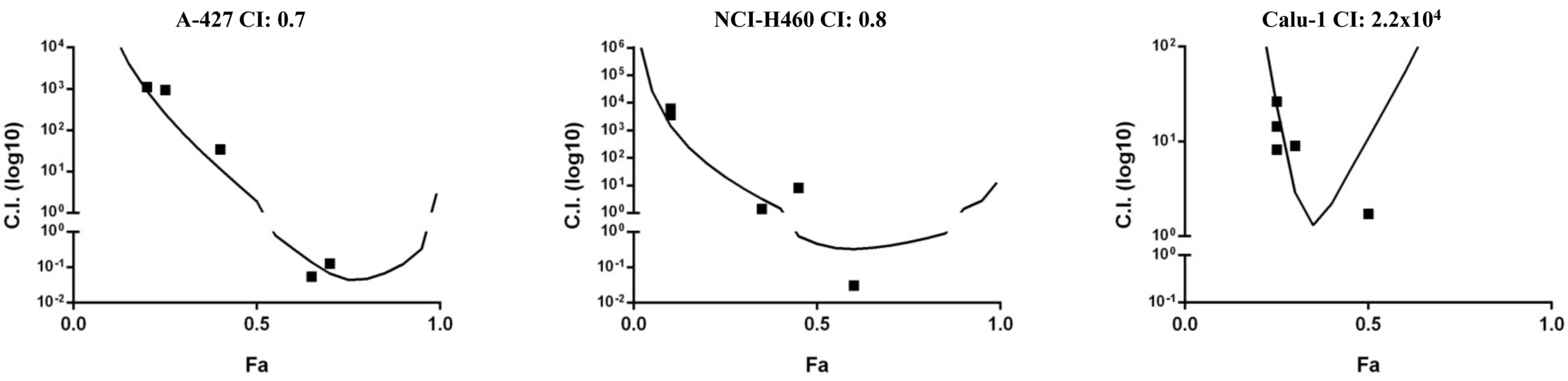

Figure S4

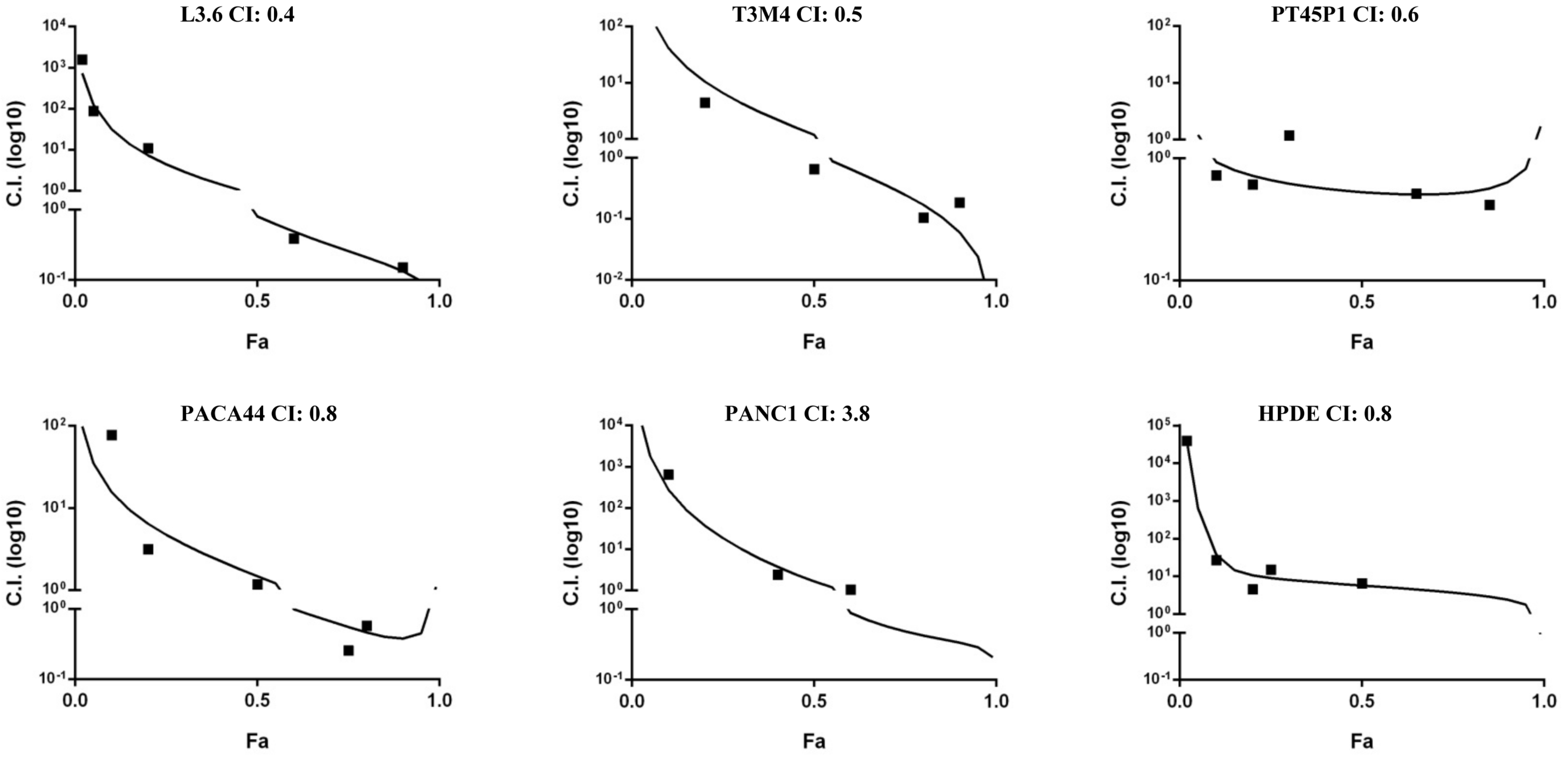

L3.6 CI: 0.4
T3M4 CI: 0.5
PT45P1 CI: 0.6
PACA44 CI: 0.8
PANC1 CI: 3.8
HPDE CI: 0.8
C.I. (log10)
Fa
0.0
0.5
1.0

Figure S5

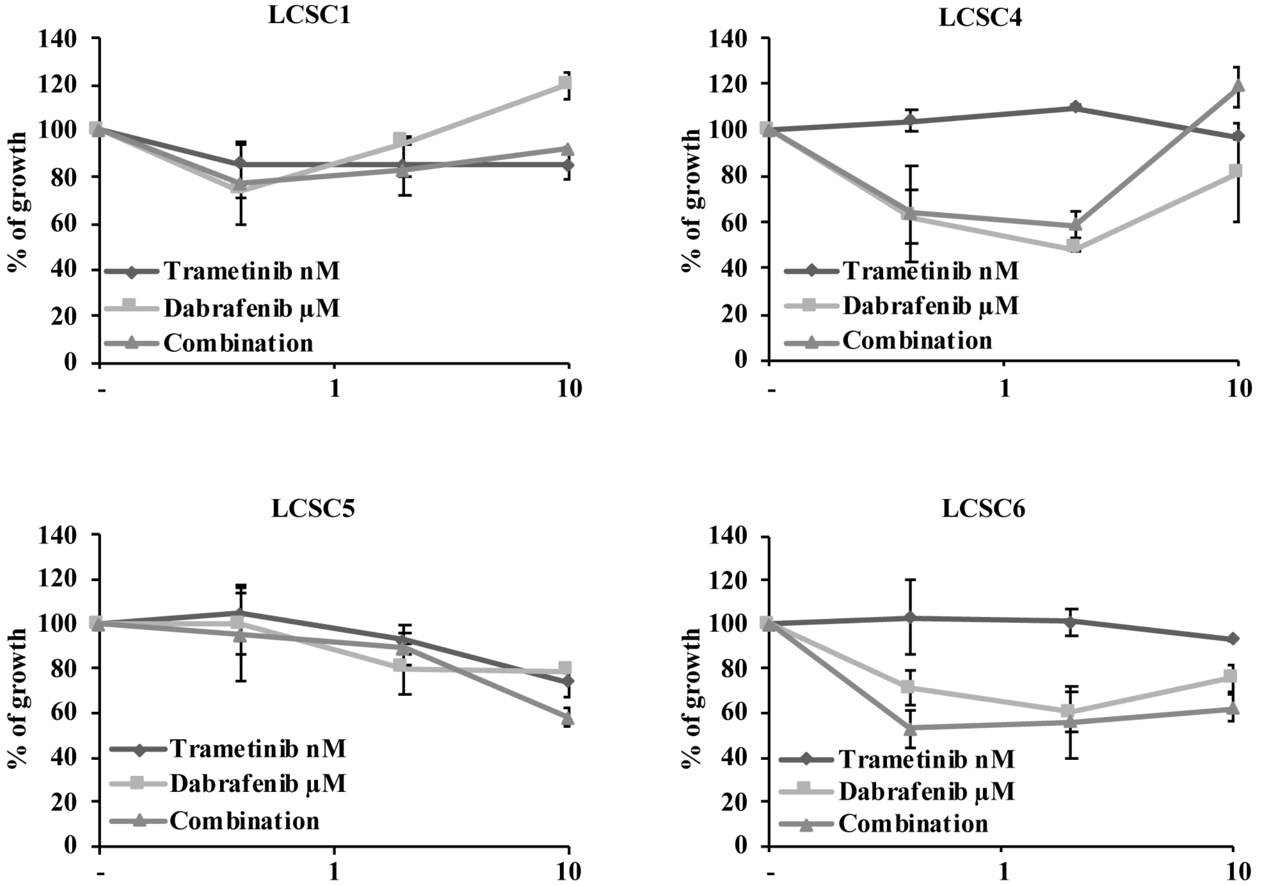
LCSC1
140
120
100
80
60
40
20
0
% of growth
Trametinib nM
Dabrafenib µM
Combination
-
1
10
LCSC4
% of growth
Trametinib nM
Dabrafenib µM
Combination
LCSC5
% of growth
Trametinib nM
Dabrafenib µM
Combination
LCSC6
% of growth
Trametinib nM
Dabrafenib µM
Combination

Figure S6

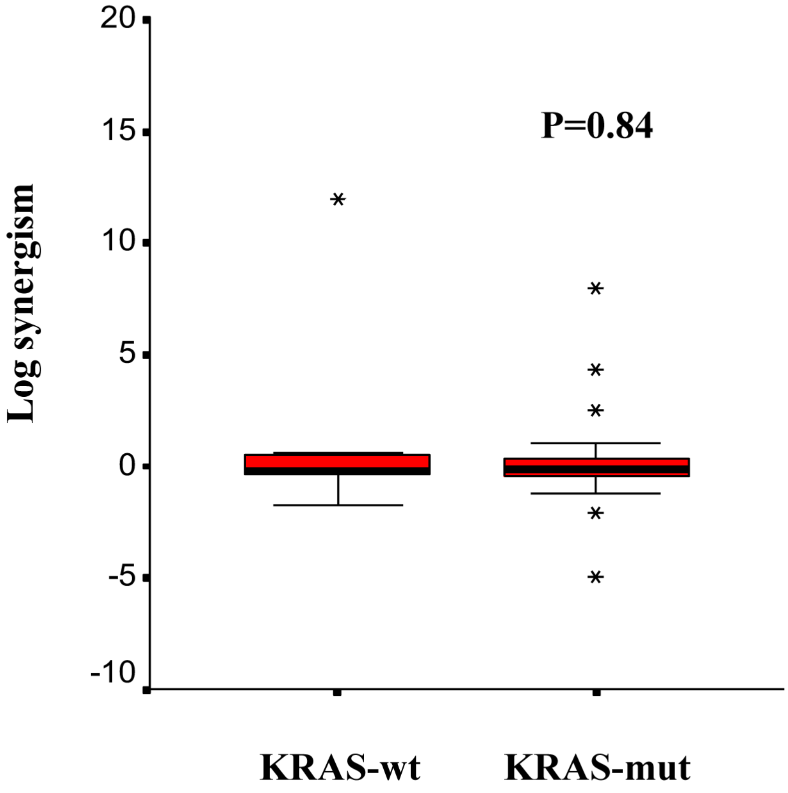

Figure S7

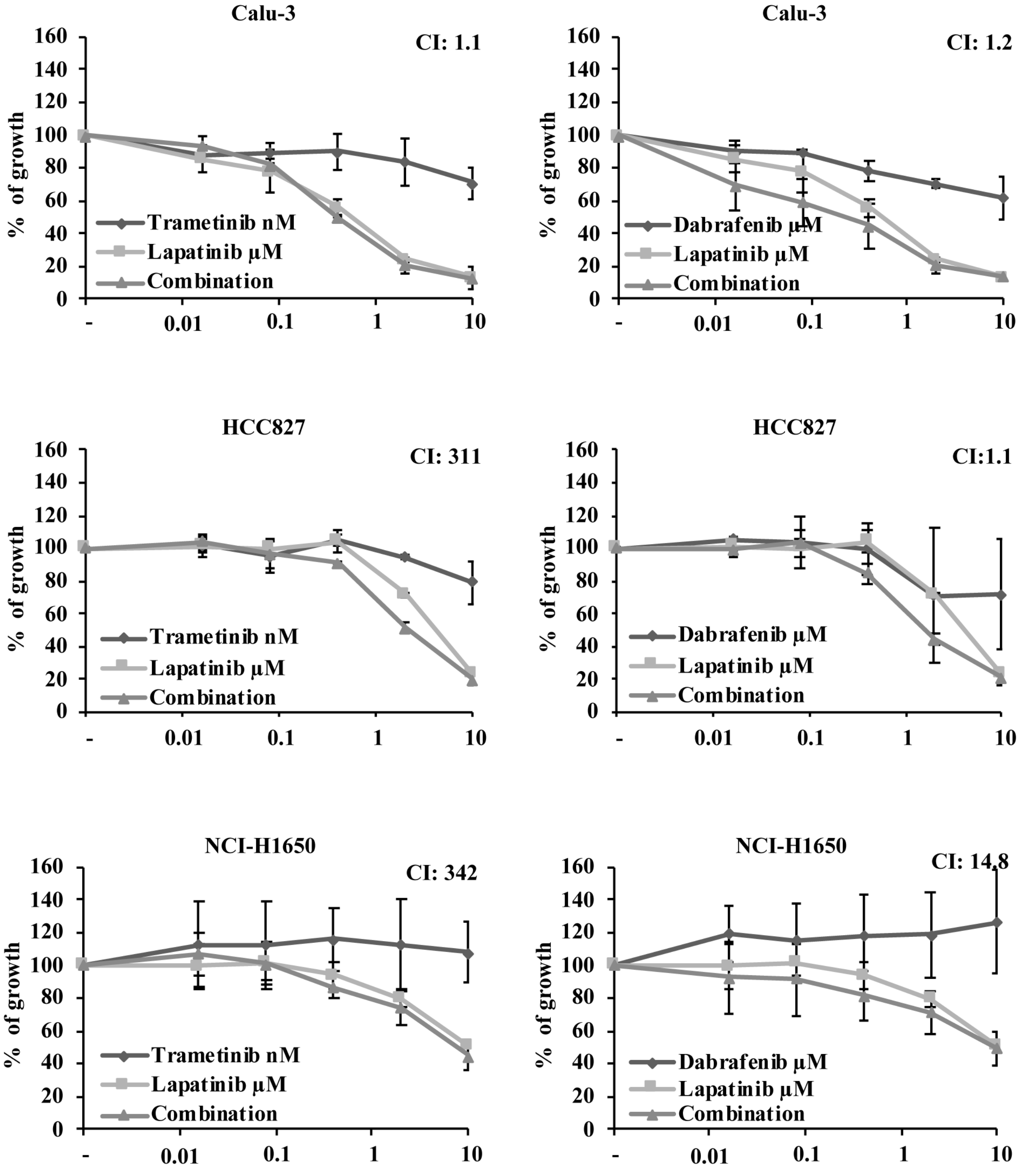
Calu-3
CI: 1.1
% of growth
Trametinib nM
Lapatinib µM
Combination
0
20
40
60
80
100
120
140
160
-
0.01
0.1
1
10
Calu-3
CI: 1.2
% of growth
Dabrafenib µM
Lapatinib µM
Combination
HCC827
CI: 311
% of growth
Trametinib nM
Lapatinib µM
Combination
HCC827
CI:1.1
% of growth
Dabrafenib µM
Lapatinib µM
Combination
NCI-H1650
CI: 342
% of growth
Trametinib nM
Lapatinib µM
Combination
NCI-H1650
CI: 14.8
% of growth
Dabrafenib µM
Lapatinib µM
Combination
